# Design, Characterization, and Hematopoietic Efficacy of a Fluorinated Pyrazolopiperidine Inclusion Complex

**DOI:** 10.3390/molecules30204047

**Published:** 2025-10-11

**Authors:** Zhanargul Koshetova, Guldana Daulet, Assel Ten, Raushan Koizhaiganova, Lyailya Baktybayeva, Tolganay Zharkynbek, Alexey Zazybin, Tulegen Seilkhanov, Nurgul Zhumanova, Valery Dembitsky, Valentina Yu

**Affiliations:** 1 Laboratory of Synthetic and Natural Medicinal Compounds Chemistry, A.B. Bekturov Institute of Chemical Sciences, 106 Sh. Ualikhanov St., Almaty 050010, Kazakhstan; zhanar.kozhetova@gmail.com (Z.K.); k_raushan@yahoo.com (R.K.); tolganay.zharkynbek@gmail.com (T.Z.); yuvkconst@gmail.com (V.Y.); 2 Department of Biophysics, Biomedicine and Neuroscience, Al-Farabi Kazakh National University, 71 Al-Farabi Ave., Almaty 050040, Kazakhstan; daulet.guldana2021@gmail.com (G.D.); lilaturap707@gmail.com (L.B.); 3 School of Chemical Engineering, Kazakh-British Technical University, 59 Tole Bi St., Almaty 050000, Kazakhstan; azazybin@yahoo.com; 4 Laboratory of Engineering Profile NMR Spectroscopy, Sh. Ualikhanov Kokshetau State University, 76 Abai St., Kokshetau 020000, Kazakhstan; tseilkhanov@mail.ru; 5 Department of Chemistry, Kazakh National Women’s Teacher Training University, 114 K1 Gogol St., Almaty 050000, Kazakhstan; n.zhumanova71@gmail.com; 6 Centre for Applied Research, Innovation and Entrepreneurship, Lethbridge Polytechnic, 3000 College Drive South, Lethbridge, AB T1K 1L6, Canada; devalery@gmail.com

**Keywords:** complex β-Cyclodextrin with 5-benzyl-7-(2-fluorobenzylidene)-2,3-bis(2-fluorophenyl)-3,3a,4,5,6,7-hexahydro-2H-pyrazolo[4,3-c]pyridine (**PPβCD**), cyclophosphamide, lymphocytopoietic-stimulating activity, myelopoietic-stimulating activity

## Abstract

A novel inclusion complex of a fluorinated pyrazolopiperidine derivative (5-benzyl-7-(2-fluorobenzylidene)-2,3-bis(2-fluorophenyl)-3,3a,4,5,6,7-hexahydro-2H-pyrazolo [4,3-c]pyridine hydrochloride, PP·HCl) with β-cyclodextrin (**PPβCD**) was designed, synthesized, and characterized as a potential therapeutic agent for chemotherapy-induced myelosuppression and lymphopenia. Encapsulation of PP within β-cyclodextrin increased aqueous solubility by approximately 3.4-fold and improved dissolution rate by 2.8-fold compared with the free compound. Structural analysis using IR, ^1H/^13C NMR, and TLC confirmed the formation of a stable 1:1 host–guest complex, and the disappearance of free PP signals further supported complete encapsulation. In vivo evaluation in a cyclophosphamide-induced myelosuppression model demonstrated that **PPβCD** accelerated hematopoietic recovery, restoring leukocyte and erythrocyte counts 35–40% faster than methyluracil, without any signs of systemic toxicity. These findings indicate that β-cyclodextrin complexation significantly enhances solubility, dissolution, and biological efficacy of the pyrazolopiperidine scaffold, supporting further preclinical development of **PPβCD** as a supportive therapy for chemotherapy-related hematological complications.

## 1. Introduction

Chemotherapy-induced myelosuppression and lymphopenia are among the most severe adverse effects of alkylating agents such as cyclophosphamide, significantly compromising immune competence, delaying treatment schedules, and reducing patient survival. Current myelostimulants—primarily nucleic acid derivatives like methyluracil (MU) and cytokine-based preparations—are limited by pyrogenicity, tumor-promoting activity, and exacerbation of autoimmune or inflammatory conditions, restricting their clinical applicability [1,2,3]. This clinical challenge has driven the search for next-generation small molecules that combine potent hematopoietic activity with improved safety profiles and chemical tractability.

The strategic incorporation of fluorine atoms into drug-like scaffolds is a widely adopted medicinal chemistry approach to modulate lipophilicity, metabolic stability, target affinity, and membrane permeability [4,5,6]. Over the past decade, organofluorine motifs have been increasingly represented among FDA-approved drugs, reflecting their utility in fine-tuning pharmacokinetic and pharmacodynamic properties [7,8]. However, fluorination must be applied judiciously, as inappropriate placement can introduce metabolic liabilities or toxic byproducts [9].

Pyrazolopiperidines constitute a privileged heterocyclic framework with diverse biological activities, including antiviral, antiarrhythmic, and analgesic effects [10,11,12]. Recent medicinal chemistry efforts have highlighted their potential as modulators of protein–ligand interactions, with clear structure–activity relationships driving optimization in antiviral and anticancer programs [13,14]. The targeted introduction of electron-withdrawing fluorine substituents into the pyrazolopiperidine scaffold can influence conformational preferences, enhance metabolic stability, and potentially improve biological target engagement. A pyrazolopiperidine scaffold was chosen over a pyrazolopyridine to reduce aromatic ring count and planarity, which are often associated with poor solubility and unfavorable pharmacokinetics [14]. The saturated piperidine ring enhances aqueous solubility, provides tunable basicity, and is recognized as a privileged scaffold in numerous clinically used drugs [10,11,12]. Several studies report bioactive pyrazolopiperidine derivatives, underscoring their translational relevance [13,15,16]. Beyond these effects, recent work has demonstrated additional activities of pyrazolopiperidine analogues, such as enzyme inhibitors and receptor modulators. For example, Genin et al. [13] described potent inhibitors of serine palmitoyltransferase that lowered ceramide levels and improved metabolic profiles in vivo, while Hu et al. [15] reviewed small-molecule inhibitors of palmitoyltransferases, including pyrazolopiperidine derivatives. Related pyrazole scaffolds, such as the 1,5-diphenyl-pyrazole series studied by Shi et al. [17], also display strong biochemical and in vivo bioactivity, further illustrating the versatility and therapeutic potential of this heterocyclic class. Taken together, these findings indicate that replacing the aromatic pyridine with a saturated piperidine ring improves physicochemical properties while maintaining—and in many cases broadening—the biological relevance of pyrazole-based heterocycles [13,15,16].

The hydrochloride salt form is a commonly adopted strategy for basic small molecules to improve solid-state stability, crystallinity, and apparent solubility, facilitating purification and formulation. Recent advances in salt synthesis methodology underscore that salt forms can greatly enhance manufacturability, stability, and dosage performance [18]. Comprehensive reviews of pharmaceutical approaches likewise emphasize that salt formation remains one of the most straightforward and effective tools for improving solubility and bioavailability for poorly soluble drug candidates [19]. However, salt stability must be carefully controlled in solid oral dosage forms, because dissociation or disproportionation can undermine product performance [20]. To further overcome these limitations and achieve additional enhancement of solubility and bioavailability, inclusion complexation with β-cyclodextrin (β-CD) offers a complementary and well-established strategy [21].

**Figure 1:**
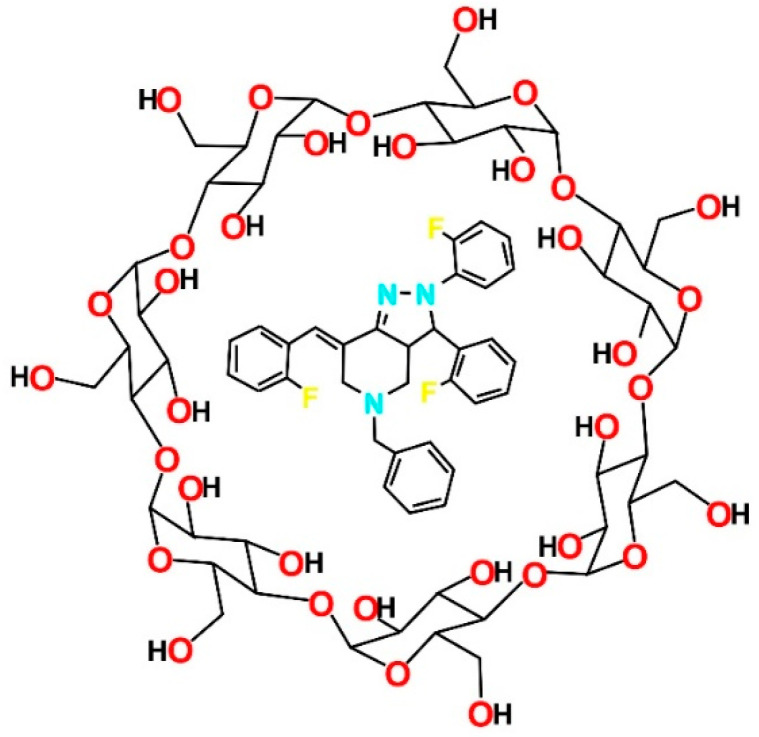
Chemical structure of the β-cyclodextrin inclusion complex of 5-benzyl-7-(2-fluorobenzylidene)-2,3-bis(2-fluorophenyl)-3,3a,4,5,6,7-hexahydro-2H-pyrazolo[4,3-c]pyridine (**PPβCD**).

The β-cyclodextrin (β-CD) inclusion complexation strategy is an established platform for improving the solubility, stability, and bioavailability of hydrophobic molecules [22,23,24,25,26]. In drug delivery, β-CD complexes have demonstrated the ability to enhance pharmacokinetic profiles, reduce local irritation in parenteral formulations [27,28], and protect labile functional groups from premature metabolism, thereby extending the systemic half-life. β-CD encapsulation, therefore, represents a well-validated formulation strategy [25]. The chemical structure of the β-CD inclusion complex of 5-benzyl-7-(2-fluorobenzylidene)-2,3-bis(2-fluorophenyl)-3,3a,4,5,6,7-hexahydro-2H-pyrazolo[4,3-c]pyridine (**PPβCD**) is shown in Figure 1.

The purpose of this study was to synthesize, spectroscopically characterize, and biologically evaluate **PPβCD**. We hypothesized that **PPβCD** would accelerate multi-lineage hematopoietic recovery in a cyclophosphamide-induced myelosuppression model while maintaining a favorable safety profile.

## 2. Results and Discussion

**Scheme 1:**
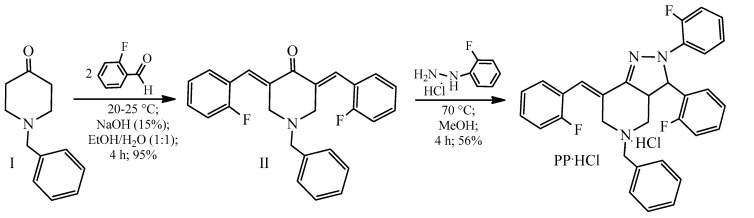
Synthesis of 5-benzyl-7-(2-fluorobenzylidene)-2,3-bis(2-fluorophenyl)-3,3a,4,5,6,7-hexahydro-2H-pyrazolo[4,3-*c*]pyridine hydrochloride.

We obtained the known 1-benzyl-3,5-bis(o-fluorobenzylidene)piperidin-4-one (II) [29] in 95% yield by condensing o-fluorobenzaldehyde with 1-benzylpiperidin-4-one I in a 2:1 molar ratio under basic conditions with 15% sodium hydroxide in ethanol (Scheme 1).

In the infrared (IR) spectrum of dienone II (Figure S1), the absorption band at 1671.36 cm^−1^ corresponds to the stretching vibrations of the carbonyl group. The C=C bonds conjugated with carbonyl absorb at 1586.37 cm^−1^, with their intensity exceeding that of the conjugated carbonyl band. The absorption at 1070.89 cm^−1^ is assigned to C-N stretching vibrations, while the band at 1148.26 cm^−1^ corresponds to C-F stretching.

The structure of the synthesized dienone II was confirmed by ^1^H and ^13^C NMR spectroscopy. The observed chemical shifts are consistent with the proposed structure. Complete spectra together with their detailed interpretation are presented in Figures S2–S6. The cyclization of 1-benzyl-3,5-bis(2-fluorobenzylidene)piperidin-4-one (II) with *o*-fluorophenylhydrazine hydrochloride yielded pyrazolopiperidine hydrochloride (PP∙HCl) in 56% yield, with a melting point of 145–146 °C. Neutralization of PP∙HCl with an aqueous potassium carbonate solution, followed by the extraction, afforded the free base PP.

**Scheme 2:**
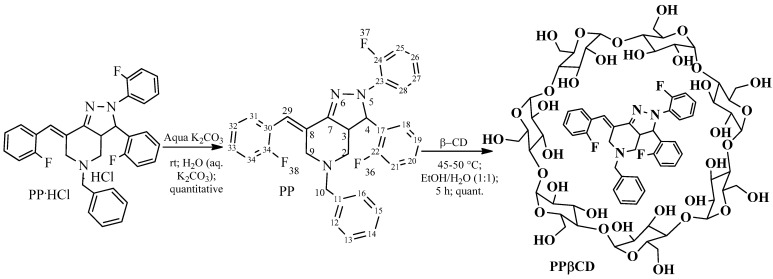
Synthesis and complexation pathway of 5-benzyl-7-(2-fluorobenzylidene)-2,3-bis(2-fluorophenyl)-3,3a,4,5,6,7-hexahydro-2H-pyrazolo[4,3-*c*]pyridine.

The inclusion complex of PP with β-cyclodextrin (β-CD) was then obtained by stirring the components in aqueous medium in a 1:1 molar ratio, which resulted in the formation of the encapsulated derivative **PPβCD**, as shown in Scheme 2. The reaction mixture was continuously stirred at 45–50 °C for 5 h, and the progress was monitored by TLC until the spot of the starting compound PP with Rf 0.49 (benzene–dioxane = 10:1) completely disappeared.

The formation of the pyrazolo[4,3-c]piperidine framework was verified by its IR spectrum (Figure S7). Characteristic absorption bands were observed at 1608.28 cm^−1^, corresponding to C=N stretching vibrations; 1589.89 cm^−1^, attributable to C=C stretching within the aromatic system; 1105.59 cm^−1^, assigned to C-F stretching; and 1027.83 cm^−1^, corresponding to C-H in-plane bending modes.

**Figure 2:**
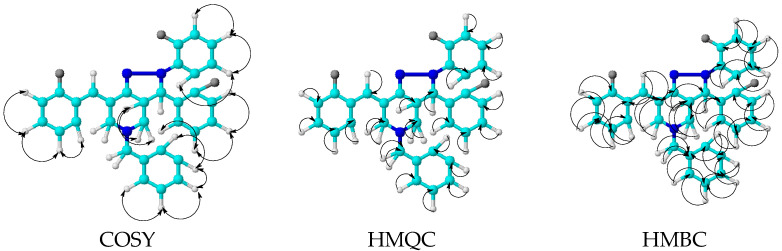
The correlation scheme in the COSY, HMQC, and HMBC spectra of 5-benzyl-7-(2-fluorobenzylidene)-2,3-bis(*2*-fluorophenyl)-3,3a,4,5,6,7-hexahydro-2H-pyrazolo[4,3-*c*]pyridine.

The ^1^H and ^13^C NMR spectra together with their assignments are provided in Figures S8–S12, which are in full agreement with the proposed structure. In the ^13^C NMR spectrum of PP (Figure S9), no resonance corresponding to a carbonyl carbon (typically near 187 ppm) is present, confirming the absence of this functional group. Instead, the C_7_ carbon is detected as a broad singlet at 161.92 ppm. Moreover, reduced intensity of signals associated with carbons at the former double-bond positions supports the occurrence of the heterocyclization. This transformation is further substantiated by the observed disruption of molecule symmetry in the spectrum. In addition, the analysis of 2D NMR spectra, including COSY, HMQC, and HMBC, revealed the expected correlations, thereby confirming the proposed structure of the compound (Figure 2).

The inclusion complex (**PPβCD**) was prepared by combining aqueous-alcoholic solutions of β-cyclodextrin and pyrazolopiperidine (PP), followed by slow evaporation of the water–ethanol mixture over 4 h at 50–55 °C.

Analysis of the IR spectra for the physical mixture (1:1), the inclusion complex, and the starting components (PP as the guest molecule and β-cyclodextrin as the host) is shown in Figure S13. In the spectrum of PP, characteristic aromatic ring vibrations were observed at 3060–3030 cm^−1^, while C-H stretching vibrations appear at 1608–1589 cm^−1^. In the inclusion complex, these bands shift slightly to 3060–3032 cm^−1^ and 1607–1577 cm^−1^, respectively, whereas no significant changes were detected in the physical mixture. Complex formation was further supported by thin-layer chromatography (TLC), which demonstrated the disappearance of the spot corresponding to free PP (Rf = 0.49) from the aqueous–alcoholic solution of the reaction mixture, indicating complete incorporation into the host structure. In addition, ^1^H and ^13^C NMR spectroscopy revealed characteristic chemical shift changes relative to the free compound, consistent with host–guest interactions. Given that PP and β-CD were reacted in equimolar amounts, the resulting product is consistent with a 1:1 inclusion complex. Similar indirect criteria, based on TLC and NMR spectral changes, have been successfully applied in published studies of cyclodextrin complexes with hyperoside [30] and with encapsulated anti-trypanosomal agents [31]. Together, these findings support the successful formation of a stable inclusion complex under the present experimental conditions. The structures of compounds I, II, and the PP–βCD inclusion complex were confirmed by IR and ^1^H/^13^C NMR spectroscopy, with full spectral data provided in the Supplementary Information (Figures S1–S15). The spectral characteristics of **PPβCD** and their description are presented in Figures S13–S17.

It should be noted that additional techniques, such as 2D NOESY NMR spectroscopy, powder X-ray diffraction, DSC, or mass spectrometry, can provide more direct structural confirmation of supramolecular inclusion. These complementary analyses are planned in the next stage of our research program in parallel with preclinical testing, to fully characterize the stability, crystalline form, and organization of the PP–βCD complex. At this stage, the combination of IR spectral shifts, TLC disappearance of free PP, and ^1^H/^13^C NMR data provides consistent and sufficient evidence for the successful formation of the inclusion complex. Toxicological assessment showed that the β-cyclodextrin complex of 5-benzyl-7-(2-fluorobenzylidene)-2,3-bis(2-fluorophenyl)-3,3a,4,5,6,7-hexahydro-2H-pyrazolo[4,3- c]pyridine) (**PPβCD**) exhibited an LC_50_ of 928,621.28 ± 160.22 μg/mL, indicating moderate toxicity comparable to MU (LC_50_ = 951,000.42 ± 201.22 μg/mL). To further substantiate the safety profile, acute toxicity assessment was performed in white mice, demonstrating that **PPβCD** has an LD50 value of 754.21 ± 12.54 mg/kg.

In the cyclophosphamide-induced myelosuppression model, **PPβCD** significantly increased bone marrow nucleated cell (NC) counts, achieving values comparable to MU. The average NC count in the **PPβCD** (73.90 ± 5.18) × 10^6^ cells, which was 2.94-fold higher than the untreated (UT) control (UT: 25.06 ± 1.01 × 10^6^ cells) and 1.33-fold higher than the placebo (PL) group (PL: 55.40 ± 2.81 × 10^6^ cells) (Table 1).

It is known that anemia affects over 45% of cancer patients prior to chemotherapy initiation and nearly 100% following treatment [32]. The cyclophosphamide-induced myelosuppression is a well-established experimental model for evaluating the myelostimulatory potential of new compounds [33]. In the present study, hematopoietic recovery was evaluated in rats subjected to cyclophosphamide-induced hemosuppression. In the **PPβCD** group, hemoglobin concentration reached 125.5 ± 3.0 g/L, comparable to the MU group (139.5 ± 12.1 g/L) and approaching the UT control (158.5 ± 16.5 g/L), while being 1.7-fold higher than the PL group (71.0 ± 6.0 g/L). Although MU is widely used in oncology therapy to counteract cyclophosphamide-induced toxic effects [34,35], the new compound **PPβCD** has demonstrated comparatively superior activity. Erythrocyte counts in the **PPβCD** group were 6.0 ± 0.3 × 10^12^/L, similar to MU and UT values (7.1 ± 1.1 × 10^12^/L) and 1.5-fold greater than PL (Table 2). Mean corpuscular hemoglobin (MCH) in the **PPβCD** group was 20.8 ± 0.6 pg, comparable to UT (19.45 ± 1.6 pg). Mean corpuscular hemoglobin concentration (MCHC) was 394.0 ± 5.3 g/L, slightly lower than UT (446.5 ± 16.5g/L) and MU (459.0 ± 22.5 g/L) but 1.5-fold higher than PL (647.0 ± 28.8 g/L) (Table 2). Mean corpuscular volume (MCV) in the **PPβCD** group was 52.8 ± 0.8 fl, exceeding both UT (43.5 ± 2.3 fl) and MU (40.8 ± 1.0 fl). Red cell distribution width (RDWsd) remained within the physiological range and was 2.4-fold higher than MU (Table 2). Thus, microscopy confirmed normochromic, normocytic erythropoiesis with normoblasts and reticulocyte release (reticulocyte index 4.9–6.8%).

Leukocytes and a stable blood leukogram are crucial for effective patient recovery [36]. Recent studies have explored various strategies for restoring leukocyte levels following cyclophosphamide-induced leukopenia, including the use of ultrasound therapy, plant-derived formulations, and synthetic agents [37,38,39,40,41,42]. In our study, the novel compound **PPβCD** exhibited notably high activity in promoting hematopoietic recovery. Restoration of leukopoiesis in the **PPβCD** group proceeded uniformly, without any disturbances in leukogram parameters. The total leukocyte count in this group (8.1 ± 1.5 × 10^9^/L), which was 1.12-fold higher than that in the MU group (7.2 ± 1.2 × 10^9^/L), approached the untreated control level (10.7 ± 1.1 × 10^9^/L) and was 2.1-fold higher than in the PL group (3.8 ± 0.9 × 10^9^/L). The neutrophil proportion in the **PPβCD** group was (32.2 ± 5.3)%, lower than that in the PL group (44.4 ± 1.61)%. The relative lymphocyte content was (66.2 ± 3.2)%, exceeding values observed in the MU, UT, and PL groups. The absolute lymphocyte count reached (5.4 ± 0.1) × 10^9^/L-3.6-fold higher than in the PL group ((1.5 ± 0.1) × 10^9^/L), 1.2-fold higher than in the MU group ((4.5 ± 0.1) × 10^9^/L), and close to the UT value ((6.7 ± 1.5) × 10^9^/L) (Table 2). Although MU is recognized as an effective therapeutic agent [36], **PPβCD** demonstrated superior efficacy in restoring both granulocytic and agranulocytic components of the leukogram. Peripheral blood smears confirmed a regenerative response, with the release of myeloblasts and a high proportion of band neutrophils into circulation.

The **PPβCD** group demonstrated a notably faster recovery of platelet levels compared to the other groups. Its platelet count reached (850.5 ± 22.0) × 10^9^/L, which was significantly higher than that of the MU group (340.2 ± 26.1) × 10^9^/L, the UT group (561.2 ± 12.2) × 10^9^/L, and the PL group (381.0 ± 19.6) × 10^9^/L. This treatment also led to a prompt restoration of the thrombocrit level (Table 2).

Cyclophosphamide remains one of the most extensively used chemotherapeutic agents, applied in the treatment of various malignancies such as lymphoma, multiple myeloma, leukemia, ovarian and breast cancers, small cell lung cancer, neuroblastoma, and sarcoma. In addition to its antineoplastic role, it serves as a potent immunosuppressant, frequently administered during bone marrow transplantation procedures. Clinically, it is also employed for managing nephrotic syndrome, granulomatosis with polyangiitis, autoimmune disorders, post-organ transplantation care, and several other conditions. Its distinct cytotoxic activity stems from its specialized metabolic pathway and subsequent inactivation by aldehyde dehydrogenase. Variations in cellular expression of this enzyme significantly impact both the drug’s therapeutic efficacy in oncology and its immunosuppressive potential [43]. However, cyclophosphamide is well-known for its suppressive effects on bone marrow, with prolonged administration potentially leading to severe marrow cell depletion. Among immune cells, T- and B-lymphocytes [44] exhibit the highest sensitivity to its cytotoxic effects. Experimental studies have successfully employed cyclophosphamide in murine models to induce leukopoiesis-depressive syndromes, facilitating research into hematopoietic recovery strategies [45].

In this context, we investigated the impact of **PPβCD** on myelopoiesis and lymphocytopoiesis using a mouse model of cyclophosphamide-induced bone marrow suppression. The compound effectively restored nucleated cell levels in the bone marrow, demonstrating activity comparable to that of MU.

The markedly elevated MCHC value observed in the placebo group (647.0 ± 28.8 g/L) reflects a hyperosmolar disturbance of the water–electrolyte balance resulting from cyclophosphamide-induced systemic toxicity. Such changes are commonly associated with severe dehydration due to gastrointestinal side effects, including diarrhea, loss of appetite, and vomiting, as well as potential renal impairment that disrupts fluid homeostasis. Because animals in this group received only the vehicle solution without therapeutic intervention, they displayed pronounced hematological abnormalities. Similar increases in MCHC have been reported in oncology models receiving cytotoxic agents. Importantly, treatment with the PPβCD complex normalized MCHC levels toward the physiological range, demonstrating its capacity to reduce systemic toxicity and support the restoration of water–electrolyte balance.

The data are presented as mean (M) ± standard deviation (SD) (n = 6 rats/group). One-way analysis of variance (ANOVA) has been used to determine statistically significant differences, and values have been considered reliable at *p* < 0.05 and F > F_crit_.

**Figure 3:**
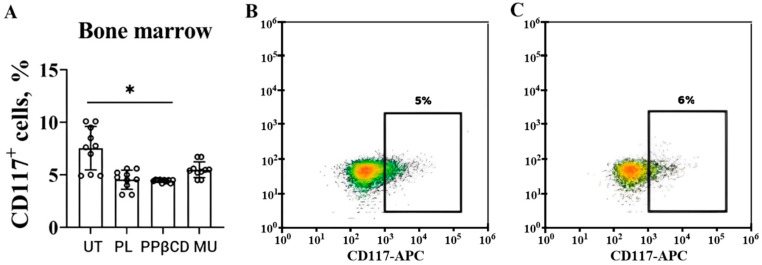
Levels of the CD117^+^ (C-kit) hematopoietic stem cells in the bone marrow: (**A**) Mean percentage of CD117^+^ (C-kit) hematopoietic stem cells in bone marrow of different groups: UT, PL, **PPβCD**, MU. (**B**) Representative flow cytometry plot for the **PPβCD** group. (**C**) Representative flow cytometry plot for the MU group. Isotope controls: (**B**)—**PPβCD** group; (**C**)—MU group. Data are expressed as M ± SD (n = 6 mice/group). * Statistical significance was evaluated using a one-way ANOVA, with differences deemed significant at *p* < 0.05 (*F* > *F*_(crit)_).

Following cyclophosphamide-induced intoxication, a marked reduction in C-kit (CD117^+^) hematopoietic stem cells (HSCs) within the bone marrow was observed. In the PL group, the CD117^+^ cell level dropped to 4.57 ± 0.6%, representing a reduction of over 40% compared to the UT group (7.62 ± 1.61%). Treatment with either **PPβCD** or MU (MU) did not produce a statistically significant improvement in CD117^+^ (C-kit) HSC counts. Mean values were comparable among the PL (4.57 ± 0.6%), **PPβCD** (4.36 ± 0.10%), and MU (6.21 ± 0.6%) groups, with all remaining 1.7–1.8 times lower than the UT group. These data, illustrated in Figure 3, indicate that while both treatments aided other hematopoietic parameters, their effect on restoring CD117^+^ stem cell levels was limited.

**Figure 4:**
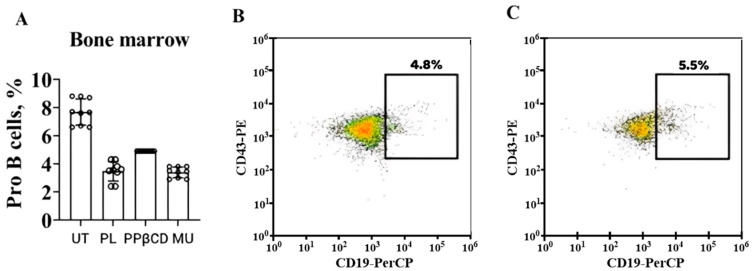
Levels of Pro-B-I and Pro-B-II lymphocytes in the bone marrow: (**A**) Mean percentage of Pro-B-I and Pro-B-II lymphocytes in the bone marrow of different groups: UT, PL, **PPβCD**, MU. (**B**) Representative flow cytometry plot for the **PPβCD** group showing 4.8% Pro-B cells. (**C**) Representative flow cytometry plot for the MU group showing 5.5% Pro-B cells. Isotype control: (**B**)—MU group, (**C**)—**PPβCD** group. Data are presented as M ± SD (n = 6 mice/group).

The bone marrow plays a pivotal role in the lymphoid pathway, supporting the proliferation and differentiation of early B-lineage cells, including Pro-B-I, Pro-B-II, Pre-B-I, Pre-B-II, and immature B-lymphocytes. Treatment with **PPβCD** markedly enhanced the recovery of Pro-B-I and Pro-B-II lymphocytes compared to MU, as reflected in the higher percentage of these cells in the bone marrow (Figure 4A). Flow cytometry profiles further confirm this effect, with the **PPβCD** group showing 4.8% Pro-B cells (Figure 4B) and the MU group showing 5.5% (Figure 4C). The ability of **PPβCD** to restore the B-lymphocyte compartment suggests a potential downstream benefit in expanding transitional B cells, which are essential for generating adequate numbers of mature B1 and B2 cells.

**Figure 5:**
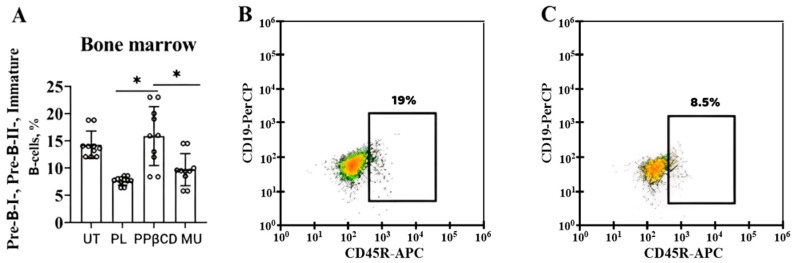
Effect of **PPβCD** on Pre-B-I, Pre-B-II, and immature B-lymphocyte levels in the bone marrow: (**A**) Mean percentage of B220^+^/CD45R^+^ CD19^+^ Pre-B-I, Pre-B-II, and immature B cells in the UT, PL, **PPβCD**, and MU groups. (**B**) Representative flow cytometry plot for the **PPβCD** group. (**C**) Representative flow cytometry plot for the PL group. Data are presented as mean ± standard deviation (SD), n = 6 mice/group. * Statistical significance was evaluated using a one-way ANOVA, with differences deemed significant at *p* < 0.05 (*F* > *F*_(crit)_).

Treatment with **PPβCD** resulted in marked restoration of B220^+^/CD45R^+^ CD19^+^ Pre-B-I, Pre-B-II, and immature B cell populations in the bone marrow. The proportion of these cells in the **PPβCD** group averaged 15.90 ± 1.34%, which was more than double that observed in the placebo group (PL, 7.66 ± 0.52%) and 1.65 times higher than in the MU (MU) group (9.61 ± 1.97%). Notably, the recovery achieved with **PPβCD** closely matched the untreated control (UT, 14.04 ± 2.01%), indicating near-complete normalization of early B-cell populations following cyclophosphamide-induced suppression (Figure 5).

**Figure 6:**
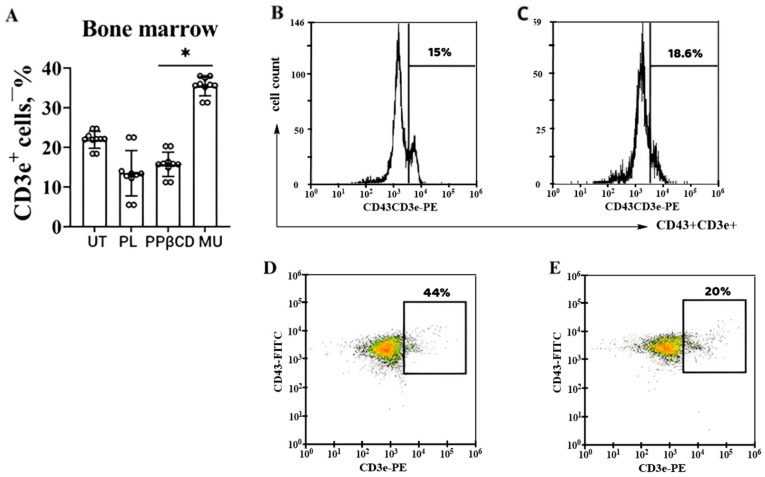
Effect of treatments on CD3e^+^ T-lymphocyte levels in mouse bone marrow: (**A**) Mean percentage (±SD, n = 6) of CD3e^+^ T-lymphocytes in the bone marrow across experimental groups: UT, PL, **PPβCD**, MU. (**B**–**E**) Representative flow cytometry plots showing the proportion of CD3e^+^ T-lymphocytes for UT (**B**), PL (**C**), **PPβCD** (**D**), and MU (**E**) groups. Isotype controls were included for each analysis. * Statistical significance was evaluated using a one-way ANOVA, with differences deemed significant at *p* < 0.05 (*F* > *F*_(crit)_).

Cyclophosphamide-induced bone marrow suppression typically delays full T-lymphocyte regeneration until around 60 days after the initial dose. Remarkably, treatment with **PPβCD** promoted detectable T-lymphocyte recovery within just 9 days. In the PL group, cyclophosphamide intoxication reduced the proportion of CD3e^+^T lymphocytes 12.06 ± 1.87%, a substantial decline compared with the UT control value of 22.01 ± 1.06%. Administration of **PPβCD** elevated this proportion to 15.73 ± 1.51%, significantly above the PL level and approaching the UT reference, although still 2.26-fold lower than that observed with MU treatment (35.60± 5.63%). Flow cytometry plots in Figure 6 illustrate these differences, showing the marked reduction in the PL group (C) versus partial restoration in the **PPβCD** group (D), and the high recovery in the MU group, consistent with the quantitative data presented in panel A.

**Figure 7:**
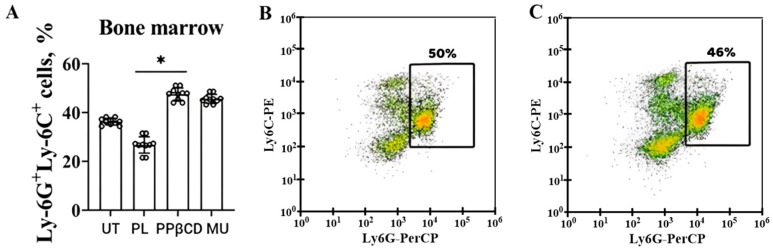
Levels of Ly-6G^+^ Ly-6C^+^ granulocytic and monocytic cells in the bone marrow: (**A**) Mean percentage of Ly-6G^+^ Ly-6C^+^ granulocytic and monocytic cells in the bone marrow of the UT, PL, **PPβCD**, and MU groups. (**B**,**C**) Representative flow cytometry dot plots showing Ly-6G^+^ Ly-6C^+^ cell populations in the **PPβCD** and MU groups, respectively. Data are presented as mean ± standard deviation (n = 6 mice/group). * Statistical significance was evaluated using a one-way ANOVA, with differences deemed significant at *p* < 0.05 (*F* > *F*_(crit)_).

Treatment with **PPβCD** markedly elevated the proportion of Ly-6G^+^ Ly-6C^+^ granulocyte and monocytic cells in the bone marrow, reaching levels comparable to those in the MU (MU) group. In the **PPβCD** group, the mean percentage was 47.55 ± 6.34%, closely matching the MU value of 45.56 ± 3.15%. This represents a 1.77-fold increase over the PL group (26.76 ± 10.14%) and a 1.31-fold increase compared with the UT group (36.28 ± 2.01%). These results, illustrated in Figure 7, indicate that **PPβCD** effectively restores and enhances granulocyte–monocyte populations following cyclophosphamide-induced suppression.

Monocytopenia is often associated with increased susceptibility to non-tuberculous mycobacterial infections, fungal pathogens, and viral illnesses, which can progress to secondary malignancies. It also elevates the risk of various hematological abnormalities [46,47], neutropenia, and opportunistic infections, including fungal involvement of mucosal tissues [48]. A prompt restoration of granulocytic and monocytic leukocytes is therefore critical for maintaining both innate and adaptive immune functions. In this study, **PPβCD** demonstrated strong efficacy in promoting the recovery of B lymphocytes as well as granulocytic and monocytic leukocytes. Remarkably, cell populations were restored within 7 days despite pronounced intoxication and myelosuppression induced by a cytostatic agent.

**Figure 8:**
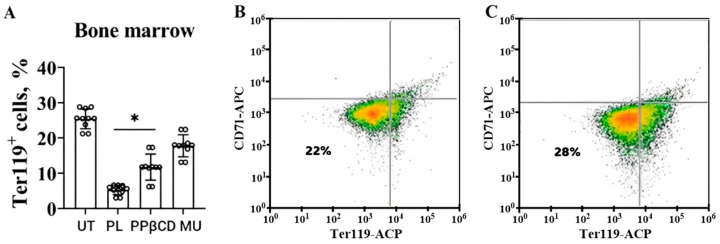
Levels of CD71^+^Ter119^+^ erythroid cells in the bone marrow: (**A**) Mean percentage of CD71^+^Ter119^+^ erythroid cells in the UT group, PL group, **PPβCD** group, and MU group. (**B**,**C**) Representative flow cytometry dot plots showing the CD71^+^Ter119^+^ cell population in **PPβCD**- and MU-treated groups. Data are expressed as M ± SD (n = 6 mice/group). * Statistical significance was evaluated using a one-way ANOVA, with differences deemed significant at *p* < 0.05 (*F* > *F*_(crit)_).

Ter119^+^ is a marker specific to the erythroid lineage, appearing on early proerythroblasts prior to their maturation into erythrocytes. Cyclophosphamide-induced intoxication led to a pronounced reduction, by approximately 77.81%, in the CD71^+^Ter119^+^ erythroid population, with the PL group showing only 5.67 ± 0.85% compared to 25.55 ± 2.60% in the UT group, a level characteristic of severe anemia. Administration of **PPβCD** significantly restored early proerythroblast counts to 17.8 ± 1.6%, representing a 3.13-fold increase over the PL group, though still lower than the activity observed with MU. These findings indicate that **PPβCD** partially reverses cyclophosphamide-induced erythroid suppression (Figure 8).

The C-kit (CD117^+^) is a type III receptor tyrosine kinase located on the surface of hematopoietic stem cells. At the early stage of differentiation, bone marrow stem cells should remain unresponsive to external physical or chemical stimuli to minimize the risk of oncogenic mutations and malignant transformation resulting from uncontrolled proliferation. Mutations in the C-kit proto-oncogene have been implicated in the formation of gastrointestinal stromal tumors, acute myeloid leukemia, melanoma, and testicular cancer [49]. In the bone marrow, during active hematopoiesis, CD117^+^ is abundantly expressed on hematopoietic stem cells, pluripotent precursors, and promyeloblasts, whereas lymphoid progenitors exhibit only minimal surface expression. Functionally, CD117^+^ serves as a differentiation cluster marker, identifying progenitor cells and regulating normal hematopoietic processes [50]. Targeted therapeutic agents that inhibit CD117^+^ expression are under development. *Imatinib*, an inhibitor of the KIT gene, was among the first introduced internationally [51], though later studies revealed its ineffectiveness in tumors harboring mutations in exon 17 of KIT, for which alternative monoclonal antibody inhibitors such as *dasatinib* and *nilotimib* are employed [52]. Notably, **PPβCD** did not increase the proportion of C-kit (CD117^+^) hematopoietic stem cells in the bone marrow following cyclophosphamide-induced myelosuppression. This lack of stimulatory effect is considered beneficial, as it reduces the risk of promoting malignant cell proliferation.

## 3. Materials and Methods

*General considerations.* The progress of the reactions and the purity of the products were monitored using TLC (thin-layer chromatography) on aluminum oxide (activity grade II), with benzene–dioxane (7:1, 10:1, *v*/*v*) as the mobile phase, and spots were visualized in iodine vapor. Product purity was confirmed using a Rapid Micro N Cube elemental analyzer and a Melting Point Apparatus Model MP490 (melting point range ≤ 1 °C). IR spectra were recorded on a Bruker Alpha-P ATR FTIR spectrometer with a diamond crystal, using KBr pellets. The ^1^H and ^13^C NMR spectra of samples II and PP were acquired on a JNM-ECA 400 (Jeol) spectrometer operating at 399.78 MHz (^1^H) and 100.53 MHz (^13^C) in deuterated chloroform (CDCl_3_) as the solvent. Chemical shifts were referenced to residual proton and carbon of CDCl_3_ (δ_H 7.26 ppm, δ_C 77 ppm at 20 °C). The compound concentration in the deuterated solvent was 10%, with no suppression of solvent signals applied. For the inclusion complex (**PPβCD**), ^1^H and ^13^C NMR spectra were recorded on a JNM-ECZ 600R spectrometer (JEOL, Tokyo, Japan) operating at 600 MHz (^1^H) and 150 MHz (^13^C) in DMSO-d_6_ solutions. Tetramethylsilane (TMS) served as the internal standard for ^1^H NMR spectra, while the solvent signal (DMSO-d_6_, 39.52 ppm relative to TMS) was used as the reference for ^13^C NMR spectra. All supporting spectral data are provided in the Supplementary Information.

*1-(Benzyl)-3,5-bis(2-fluorobenzylidene)piperidone-4 (II).* A solution of sodium hydroxide (4.2 g, 0.105 mol) was prepared in 40 mL of water and 40 mL of ethanol. To half of this freshly prepared alkaline mixture, 1-benzylpiperidin-4-one (I) (4.0 g, 0.021 mol) and o-fluorobenzaldehyde (5.3 g, 0.043 mol) were added. After stirring for 15 min, the remaining half of the water–ethanol NaOH solution was added. The reaction mixture was stirred continuously for 4 h at 20–25 °C. The resulting precipitate was collected by filtration, washed with water, dried, and recrystallized from hexane to yield 1-benzyl-3,5-bis(2-fluorobenzylidene)piperidone-4 (II) as a pale solid (8.0 g, 95%). Rf = 0.43 (eluent: benzene–dioxane = 7:1), mp. 150–151 °C.

The elemental analysis for C_26_H_21_NOF_2_ (calcd/found, %): C, 77.79/77.79; H, 5.27/5.23; F, 9.46/9.37; N, 3.49/3.21; O, 3,99/3,92. IR (KBr, cm^−1^): 1671.36 (C=O), 1070.89 (C-N), 1586.37 (C=C), 1148.26 (C-F) (Figure S1). ^1^H NMR (399.78 MHz, CDCl_3_): δ 3.64–3.82 (m, 6H, 2 -CH_2_-, piperidonic, -CH_2_-), 6.97–7.36 (m, 13H, -ArH), 7.73 and 7.91 (s, 2H, 2 >C=CH-) (Figure S2). ^13^C NMR (100.53 MHz, CDCl_3_): δ 54.15 (C_2_, C_6_), 60.70, 61.49 (C_7_), 115.88, 116.88 (C_17_, C_25_, aromatic), 123.33, 123.94 (C_15_, C_23_, aromatic), 126.23 (C_9_–C_13_, aromatic), 127.34, 128.48, 129.02, 129.84 (C_19_, C_20_, C_27_, C_28_, aromatic), 130.19, 130.90 (C_14_, C_22_, olefinic), 134.33 (C_3_, C_5_), 135.01, 135.31, 137.69 (C_8_, C_18_, C_26_, aromatic), 159.73, 161.48, 162.23, 163.93 (C_16_, C_24_, fluorinated aromatic), and 187.42 (C_4_, carbonyl) (Figure S3).

*5-Benzyl-7-(2-fluorobenzylidene)-2,3-bis(2-fluorophenyl)-3,3a,4,5,6,7-hexahydro-2H-pyrazolo [4,3-c]pyridine (PP)*. A mixture of *o*-fluorophenylhydrazine hydrochloride (0.49 g, 0.003 mol) and 1-(benzyl)-3,5-bis(2-fluorobenzylidene)piperidone-4 (II) (1.20 g, 0.003 mol) in methanol (15 mL) was stirred at 70 °C for 4 h. The solvent was removed under reduced pressure, and the residue was recrystallized from methanol to give the hydrochloride salt of PP (0.92 g, 56%) as a solid, mp 145–146 °C. Neutralization of the aqueous hydrochloride solution with potassium carbonate followed by extraction with chloroform afforded PP in quantitative yield (Rf 0.49, benzene–dioxane = 10:1).

Elemental analysis for C_32_H_26_N_3_F_3_ (calcd/found, %): C, 75.43/75.39; H, 5.14/5.35; F, 11.18/12.01; N, 8.25/8.29. IR (KBr, cm^−1^): 1608.28 (C=N), 1589.89 (C=C), 1105.59 (C-F), 1027.83 (C-N) (Figure S7). ^1^H NMR (399.78 MHz, CDCl_3_): δ 2.63–2.68 (m, 1H, -CH_2_-, piperidonic), 3.21–3.38 (m, 1H, -CH_2_-,piperidonic), 3.37–3.40 (m, 1H, -CH_2_-, piperidonic), 3.42–3.51 (m, 1H, -CH_2_-, piperidonic), 3.64–3.74 (m, 2H, -CH_2_-), 3.89–3.93 (m, 1H, -CH_2_-, piperidonic), 5.26–5.30 (m, 1H, -CH<), 6.98–7.27 (m, 15H, -ArH), 7.38 (s, 1H, -ArH), 7.62–7.65 (m, 1H, -ArH) (Figure S8). ^13^C NMR (CDCl_3_, δ, ppm): 53.66 (C_3_), 54.47 (C_9_), 55.48 (C_2_), 61.76 (C_10_), 65.78 (C_4_-N), 115.57, 116.93, 119.59, 120.78, 123.76, 124.50, 124.81, 126.76, 127.37, 128.41, 129.04, 129.49, 129.61, 130.83, 133.93, 137.62 (aromatic carbons C_11_–C_16_, C_32_–C_35_, C_19_–C_23_, C_25_–C_28_, C_29_, C_30_), 152.79, 156.45, 159.43 (C-F: C_18_, C_24_, C_31_); 161.92 (C_7_) (Figure S9).

*Inclusion Complex of 5-Benzyl-7-(2-fluorobenzylidene)-2,3-bis(2-fluorophenyl)-3,3a,4,5,6,7-hexahydro-2H-pyrazolo [4,3-c]pyridine (*PP*) with β-Cyclodextrin (***PPβCD***)*. A solution of PP (0.30 g, 0.0005 mol) in ethanol (30 mL) was mixed with a solution of *β*-cyclodextrin (0.68 g, 0.0005 mol) in distilled water (30 mL). The mixture was then transferred to an oven, and the solvents evaporated at 50–55 °C for 4 h. The resulting inclusion complex **PPβCD** was obtained as a white powder (0.98 g, quantitative yield), decomposing above 240 °C.

Elemental analysis for C_74_H_96_O_35_N_3_F_3_ (calcd/found, %): C, 54.05/54.95; H, 5.88/5.63; F, 3.47/3.39; N, 2.56/2.56.

*Acute toxicity assessment in the crustacean Artemia salina.* The acute toxicity of the **PPβCD** compound was evaluated using the crustacean *Artemia Salina* as the test organism [53,54,55]. *A. Salina* cysts were stored at −20 °C until use. For hatching, 1.11 g of cysts were incubated in a shallow rectangular glass vessel (20 cm × 10 cm × 5 cm) containing 3.3% artificial seawater. A plastic divider with multiple 2mm diameter holes separated the vessel into two unequal compartments. The cysts and 0.08 g of yeast were introduced into the darker compartment, while the smaller, illuminated compartment was exposed to a tungsten lamp and continuously aerated. After 24 h, hatched *A. salina* cysts were transferred into fresh artificial seawater and maintained for an additional 24 h under the same light aeration conditions. Phototropic nauplii were collected from the illuminated side using a pipette. Test solutions of **PPβCD** and MU were prepared in three concentrations: 10,000 μg/mL, 500,000 μg/mL, and 1,000,000 μg/mL. Compounds were dissolved in 3.3% artificial seawater and sterilized by filtration through 0.22 μm membrane filters in a laminar flow hood. Aliquots were dispensed into sterile 10 mL bottles, with five replicates prepared for each dose and the control group.

For toxicity assessment, ten *A. Salina* nauplii (larvae) were transferred into each sample vial using 230 mm glass Pasteur pipettes (cat. D812, Poulten & Graf Ltd., Barking, UK). Nauplii were counted macroscopically within the pipette stem under Optic × 3.5-420L-Proff binoculars (Foshan, Guangdong, China). A drop of dry yeast suspension (3 mg in 5 mL artificial seawater) was added to each vial as feed. All vials were maintained under continuous light. After 24 h of exposure, the numbers of dead and live larvae were recorded using the same binocular microscope. Mortality percentage at each test concentration and in the control group was calculated according to Equation (1):

(1) $$Mortality\left(\%\right)=\frac{r}{n}\times 100$$

where *r* is the number of dead nauplii and *n* is the total number of *A. salina* in each vial.

The lethal concentration (LC_50_) was determined using Microsoft Excel 2010. Toxicity classification was defined as follows: highly toxic (LC_50_ = 0–500,000 µg/mL), moderate toxicity (LC_50_ = 500,000–1,000,000 µg/mL), and low or non-toxic (LC_50_ > 1,000,000 µg/mL).

*Acute toxicity assessment in white mice.* The study of acute toxicity was carried out on healthy outbred laboratory white mice weighing 19–24 g, aged 8–11 weeks, of both sexes. The animals were divided into two complexes of groups (each complex consisted of several groups, with six animals in each group), experimental and control, with the administration of the reference drug methyluracil. The test compound **PPβCD** was dissolved in physiological saline and administered intraperitoneally in increasing concentrations, in a volume not exceeding 0.05 mL, administered once. Observation of the animals was carried out for 48 h to record systemic toxic manifestations in the form of changes in physiological signs (diarrhea, dyspnea, apnea, tremor, seizures), behavioral reactions, and locomotor activity. Daily food and water intake by the mice and body weight were also recorded. Based on the results of the study, the lethal dose (LD_50_) was calculated. The approval of the Local Ethics Committee is included in Supporting Information.

*Hemostimulating Activity Assay*. To hemostimulating activity of **PPβCD** was evaluated in the peripheral blood of 24 adult virgin female albino rats (12–15 weeks old, 210–280 g). Animals were bred and housed in a pathogen-free facility under controlled temperature (21–23 °C) in standard polypropylene cages with free access to standard feed and water.

Animals were divided into four groups (n = 6 each): UT, PL, **PPβCD**, MU.

Cyclophosphamide (Baxter Oncology GmbH, Halle, Germany) was dissolved in 0.9% physiological saline and administered intramuscularly to the PL, **PPβCD**, and MU groups at a dose of 40 mg/kg (0.25–0.3 mL of 4% solution) at 09:00 on days 1, 2, and 3. From days 6 to 8, at 09:00, the following intramuscular injections were administered:

- **PPβCD** group: **PPβCD** in 0.9% saline, 10 mg/kg (0.20–0.25 mL of 1% solution);
- MU group: MU in 0.9% saline, 10 mg/kg (0.20–0.25 mL of 1% solution);
- PL group: 0.9% saline, 10 mg/kg (0.20–0.25 mL);
- UT group: no injections.

On day 15 (seven days after the last injection), at 09:00, blood was collected from the ophthalmic vein under light ketamine/xylazine anesthesia (91.0 mg/kg ketamine/9.1 mg/kg xylazine). Food was withdrawn 12 h before blood sampling. Blood was collected into sterile hematology tubes (VF-052SDK) containing EDTA (K2, 2 mL) [51]. Hematological parameters were determined using an automated hematology blood analyzer (Abacus Junior VET, Diatron, Denmark). For leukogram assessment, blood smears were prepared, stained with Giemsa [56], and examined under an immersion microscope (SA3300S, magnification 7 × 100) (Histo-Line Laboratories, Dusseldorf, Germany). A total of 100 leukocytes were counted per smear, and relative counts were converted to absolute values.

*Myelostimulating Activity Assay.* The myelostimulating activity of **PPβCD** was evaluated in female mice (5–7 weeks old, 18–23 g). Animals were bred and maintained in a pathogen-free facility under controlled temperature (21–23 °C) in standard polypropylene cages with free access to standard feed and water.

Mice were randomly assigned to four groups (n = 9 per group): UT, PL, **PPβCD**, and MU.

Cyclophosphamide (Baxter Oncology GmbH, Germany) was dissolved in 0.9% physiological saline and administered intramuscularly to the PL, **PPβCD**, and MU groups at a dose of 100 mg/kg (0.05 mL of 4% solution), at 09:00 on days 1, 3, and 5. On days 8, 10, and 12, at 09:00, the following treatments were administered intramuscularly:

- **PPβCD** group: **PPβCD** in 0.9% saline, 10 mg/kg (0.05 mL of 0.4% solution);
- MU group: MU in 0.9% saline, 10 mg/kg (0.05 mL of 0.4% solution);
- PL group: 0.9% saline, 0.05 mL;
- UT group: no injections.

On day 15 (3 days after the last treatment), animals were anesthetized with ketamine/xylazine (87.5 mg/kg ketamine/12.5 mg/kg xylazine) and euthanized by cervical dislocation.

*Cytometric Analysis of Bone Marrow Cells.* Phosphate-buffered saline (PBS, pH 7.4) was prepared by dissolving NaH_2_PO_4_ and NaCl in the purified water and adjusting the pH with NaOH. The column buffer was prepared by adding 0.5% PBS and 0.002 M EDTA to PBS. Both solutions were sterile-filtered through 0.22 μm membrane filters and stored at 4–8 °C until use.

Following euthanasia, muscles, ligaments, and tendons were surgically removed from the hind limb bones, and bone marrow was flushed with PBS using a 10 mL syringe. The contaminated erythrocytes were lysed with a solution containing 0.83% NH_4_Cl, 0.1% KHCO_3_, and 0.003% EDTA (pH 7.2–7.4) for 10 min at room temperature. The remaining cells were washed, passed through 30 μm pre-separation filters (MilteNY Biotec, Bergisch Gladbach, Germany), and counted. Cell viability was assessed using trypan blue.

For immunophenotyping, 1 × 10^6^ cells were incubated with the following mouse monoclonal antibodies (mAbs) according to manufacturer protocols: PerCP-labeled anti-Ly6G, PE-labeled anti-Ly6C, PE-labeled anti-CD43, PE-labeled anti-CD19, PE-labeled anti-CD3e, APC-labeled anti-CD71, PerCP-labeled anti-Ter119, PE-labeled anti-CD117, and APC-labeled anti-B220/CD45R. After 20 min incubation at room temperature (20–21 °C) in the dark, cells were fixed with the fixation solution (cat. no. 554722, BD Bioscience, San Diego, CA, USA), washed with PBS, resuspended in the flow solution, and analyzed immediately on a FACSCalibur flow cytometer (BD Biosciences) using the CellQuest Pro software (version 5.1) (BD Bioscience, San Diego, CA, USA). Instrument settings were established using unstained cells, single-stained controls, and fluorescence-minus-one (FMO) controls.

Data were analyzed using Microsoft Excel and the Statistics 6.0 software. Results are expressed as M ± SD for n = 9 mice per group. Statistical significance was determined using one-way ANOVA, with *p* < 0.05 and *F* > *F*_crit_ considered significant.

## 4. Conclusions

The β-CD inclusion complex of the fluorinated pyrazolopiperidine **PPβCD** exhibited marked efficacy in restoring hematopoietic and immune cell populations in a cyclophosphamide-induced myelosuppression model. Enhanced solubility and stability, conferred by β-CD complexation, likely contributed to the favorable in vivo activity and safety profile. Compared with MU, **PPβCD** achieved faster normalization of peripheral blood parameters without evidence of systemic toxicity. These results support further development of this complex as a candidate therapeutic for the supportive management of chemotherapy-induced hematologic complications. Future work should address pharmacokinetic profiling, long-term safety assessment, and potential combinational use with existing antineoplastic regimens.

## Figures and Tables

**Table 1:** Bone marrow cellularity in different treatment groups.

Group	Average Nucleated Cells (×10^6^)	*p*-Value vs. PPβCD	F-Test (F > F_crit_)
UT	25.1 ± 7.4	P_UT-PPβCD_ = 0.00006	94.66 > 5.98
MU	57.4 ± 4.2	P_MU-PPβCD_ = 0.04	6.05 > 5.98
**PPβCD**	73.9 ± 5.1	-	-
PL	61.3 ± 2.8	P_PL-PPβCD_ = 0.01	10.36 > 5.98

**Table 2:** Peripheral blood hemogram indicators.

	1	2	3	4	
Groups	PPβCD	MU	PL	UT	*p*, F
WBC, ×10^9^/L	8.1 ± 1.5 *	7.2 ± 1.2	3.8 ± 0.9	10.7 ± 1.1	*
LYM, ×10^9^/L	5.4 ± 0.1 *	4.5 ± 0.1	1.5 ± 0.1	6.7 ± 1.5	**
NEU, ×10^9^/L	2.6 ± 0.0	2.1 ± 0.6	1.6 ± 0.1	3.1 ± 0.7	***
MON, ×10^9^/L	0.1 ± 0.0	0.3 ± 0.0	0.47 ± 0.2	0.3 ± 0.0	
EO, ×10^9^/L	0.0 ± 0.0	0.2 ± 0.0	0.0 ± 0.0	0.4 ± 0.0	
BAS, ×10^9^/L	0.0 ± 0.0	0.0 ± 0.0	0.0 ± 0.0	0.2 ± 0.0	
LYM, %	66.2 ± 3.2	62.8 ± 1.7	40.6 ± 1.4	62.4 ± 3.1	****
NEU, %	32.2 ± 5.3	29.8 ± 0.6	44.4 ± 1.6	29.3 ± 1.0	*****
MON, %	1.6 ± 0.2	4.2 ± 0.7	12.2 ± 0.9	2.7 ± 0.0	******
EO, %	0.0 ± 0.0	2.8 ± 0.0	2.0 ± 0.0	3.5 ± 0.8	
BAS, %	0.0 ± 0.0	0.4 ± 0.0	0.8 ± 0.0	3.0 ± 0.0	
RBC, ×10^12^/L	6.0 ± 0.3	7.4 ± 1.1	4.0 ± 1.64	7.1 ± 1.1	
HGB, g/L	125.5 ± 3.0	139.5 ± 12.1	71.0 ± 6.0	158.5 ± 16.5	*******
HCT, %	31.9 ± 1.2 *	30.2 ± 2.3	11.0 ± 0.31	36.9 ± 3.2	
MCV, fL	52.8 ± 0.8 *	40.8 ± 1.0	26.9 ± 1.6	43.5 ± 2.3	
MCH, pg	20.8 ± 0.6	18.7 ± 1.0	17.4 ± 0.0	19.4 ± 1.6	
MCHC, g/L	394.0 ± 5.3	459.0 ± 22.5	647.0 ± 28.8	446.5 ± 16.5	
RDWsd, fL	24.7 ± 0.4	10.1 ± 0.0	11.5 ± 0.0	19.8 ± 4.6	
RDWcv	12.85 ± 0.0	17.0 ± 1.0	31.2 ± 0.3	20.9 ± 2.0	
PLT, ×10^9^/L	850.0 ± 22.0 *	340.2 ± 26.1	381.0 ± 19.6	561.2 ± 12.2	

* (P1-2 = 0.02 F > Fcrit, 40.5 > 18.51), (P1-3 = 0.001 F > Fcrit, 924.5 > 18.51); ** (P1-2 = 0.02 F > Fcrit, 40.5 > 18.51), (P1-3 = 0.0006 F > Fcrit, 1521 > 18.51); *** (P1-2 = _H_._3_.), (P1-3 = 0.01 F > Fcrit, 50 > 18.51); **** (P1-2= _H_._3_.), (P1-3 = 0.001 F > Fcrit, 578 > 18.51); ***** (P1-2 = 0.03 F > Fcrit, 288 > 18,51), (P1-3 = 0.001 F > Fcrit, 7442 > 18.51); ****** (P1-2 = 0.002 F > Fcrit, 338 > 18.51), (P1-2 = 0.0001 F > Fcrit, 5618 > 18.51); ******* (P1-3 = 0.00003 F > Fcrit, 32,768 > 18.51).

## Data Availability

The original contributions presented in this study are included in the article/Supplementary Materials. Further inquiries can be directed to the corresponding author.

## Supplementary Materials

The following supporting information can be downloaded at: https://www.mdpi.com/article/10.3390/molecules30204047/s1, Figure S1: IR (KBr, ν, cm^−1^) spectrum of 1-benzyl-3,5-bis(2-fluorobenzylidene)piperidone-4 (II); Figure S2: ^1^H NMR spectrum of 1-benzyl-3,5-bis(2-fluorobenzylidene)piperidone-4 (II) in CDCl_3_; Figure S3: ^13^C NMR spectrum of 1-benzyl-3,5-bis(2-fluorobenzylidene)piperidone-4 (II) in CDCl_3_; Figure S4: NMR ^1^H-^1^H COSY NMR spectrum of 1-benzyl-3,5-bis(2-fluorobenzylidene)piperidone-4 (II) in CDCl_3_; Figure S5: NMR ^1^H-^13^C HMQC NMR spectrum of 1-benzyl-3,5-bis(2-fluorobenzylidene)piperidone-4 (II) in CDCl_3_; Figure S6: NMR ^1^H-^13^C HMBC NMR spectrum of 1-benzyl-3,5-bis(2-fluorobenzylidene)piperidone-4 (II) in CDCl_3_; Figure S7: IR (KBr, ν, cm^−1^) spectrum of 5-(benzyl)-7-(2-fluorobenzylidene)-2,3-bis(2-fluorophenyl)-3,3a,4,5,6,7-hexahydro-2H-pyrazolo[4,3-c]pyridine (PP); Figure S8: ^1^H NMR spectrum of 5-(benzyl)-7-(2-fluorobenzylidene)-2,3-bis(2-fluorophenyl)-3,3a,4,5,6,7-hexahydro-2H-pyrazolo[4,3-c]pyridine (PP) in CDCl_3_; Figure S9: ^13^C NMR spectrum of 5-(benzyl)-7-(2-fluorobenzylidene)-2,3-bis(2-fluorophenyl)-3,3a,4,5,6,7-hexahydro-2H-pyrazolo[4,3-c]pyridine (PP) in CDCl_3_; Figure S10: NMR ^1^H-^1^H COSY NMR spectrum of 5-(benzyl)-7-(2-fluorobenzylidene)-2,3-bis(2-fluorophenyl)-3,3a,4,5,6,7-hexahydro-2H-pyrazolo[4,3-c]pyridine (PP) in CDCl_3_; Figure S11: NMR ^1^H-^13^C HMQC NMR spectrum of 5-(benzyl)-7-(2-fluorobenzylidene)-2,3-bis(2-fluorophenyl)-3,3a,4,5,6,7-hexahydro-2H-pyrazolo[4,3-c]pyridine (PP) in CDCl_3_; Figure S12: NMR ^1^H-^13^C HMBC NMR spectrum of 5-(benzyl)-7-(2-fluorobenzylidene)-2,3-bis(2-fluorophenyl)-3,3a,4,5,6,7-hexahydro-2H-pyrazolo[4,3-c]pyridine (PP) in CDCl_3_; Figure S13: IR (KBr, ν, cm^−1^) spectrum of the physical mixture (1:1), complex (PPβCD) and the starting components (PP and β-cyclodextrin); Figure S14: ^1^H NMR spectrum of Complex of 5-(benzyl)-7-(2-fluorobenzylidene)-2,3-bis(2-fluorophenyl)-3,3a,4,5,6,7-hexahydro-2H-pyrazolo[4,3-c]pyridine with β-CD (PPβCD) in DMSO-d_6_; Figure S15: ^13^C NMR spectrum of Complex of 5-(benzyl)-7-(2-fluorobenzylidene)-2,3-bis(2-fluorophenyl)-3,3a,4,5,6,7-hexahydro-2H-pyrazolo[4,3-c]pyridine with β-CD (PPβCD) in DMSO-d_6_; Figure S16: IR (KBr, ν, cm^−1^) spectrum of β-cyclodextrin; Figure S17: IR (KBr, ν, cm^−1^) spectrum of β-cyclodextrin with 5-benzyl-7-(2-fluorobenzylidene)-2,3-bis(2-fluorophenyl)-3,3a,4,5,6,7-hexahydro-2H-pyrazolo[4,3-c]pyridine (PPβCD); The Approval of the Local Ethics Committee.

## Abbreviations

The following abbreviations are used in this manuscript:

**PPβCD**	Complex β-Cyclodextrin with 5-benzyl-7-(2-fluorobenzylidene)-2,3-bis(2-fluorophenyl)-3,3a,4,5,6,7-hexahydro-2H-pyrazolo[4,3-c]pyridine
PP	1-Benzyl-3,5-bis(2-fluorobenzylidene)piperidone-4
CD	Cyclodextrin
TLC	Thin-layer chromatography
MU	Methyluracil
PL	Placebo
UT	Untreated
PBS	Phosphate-buffered saline
EDTA	Ethylenediaminetetraacetic acid
NC	Nucleated cell count
WBC	White blood cell count
LYM	Absolute/relative lymphocyte count
NEU	Absolute/relative neutrophil count
MON	Absolute/relative monocyte count
EO	Absolute/relative eosinophil count
BAS	Absolute/relative basophil count
RBC	Red blood cell count
HGB	Hemoglobin
HCT	Hematocrit
MCV	Mean corpuscular volume
MCH	Mean corpuscular hemoglobin
MCHC	Mean corpuscular hemoglobin concentration
RDWsd	Red cell distribution width—standard deviation
RDWcv	Red cell distribution width—coefficient of variation
PLT	Total platelet volume
