# Supplementary material for: Design, Characterization, and Hematopoietic Efficacy of a Fluorinated Pyrazolopiperidine Inclusion Complex
Source: Molecules. 2025 Oct 11;30(20):4047. doi: 10.3390/molecules30204047 (PMC12566042; doi:10.3390/molecules30204047)
Supplement: Supplementary file 1 [file molecules-30-04047-s001.zip › molecules-3861245-supplementary.pdf]

SUPPORTING INFORMATION

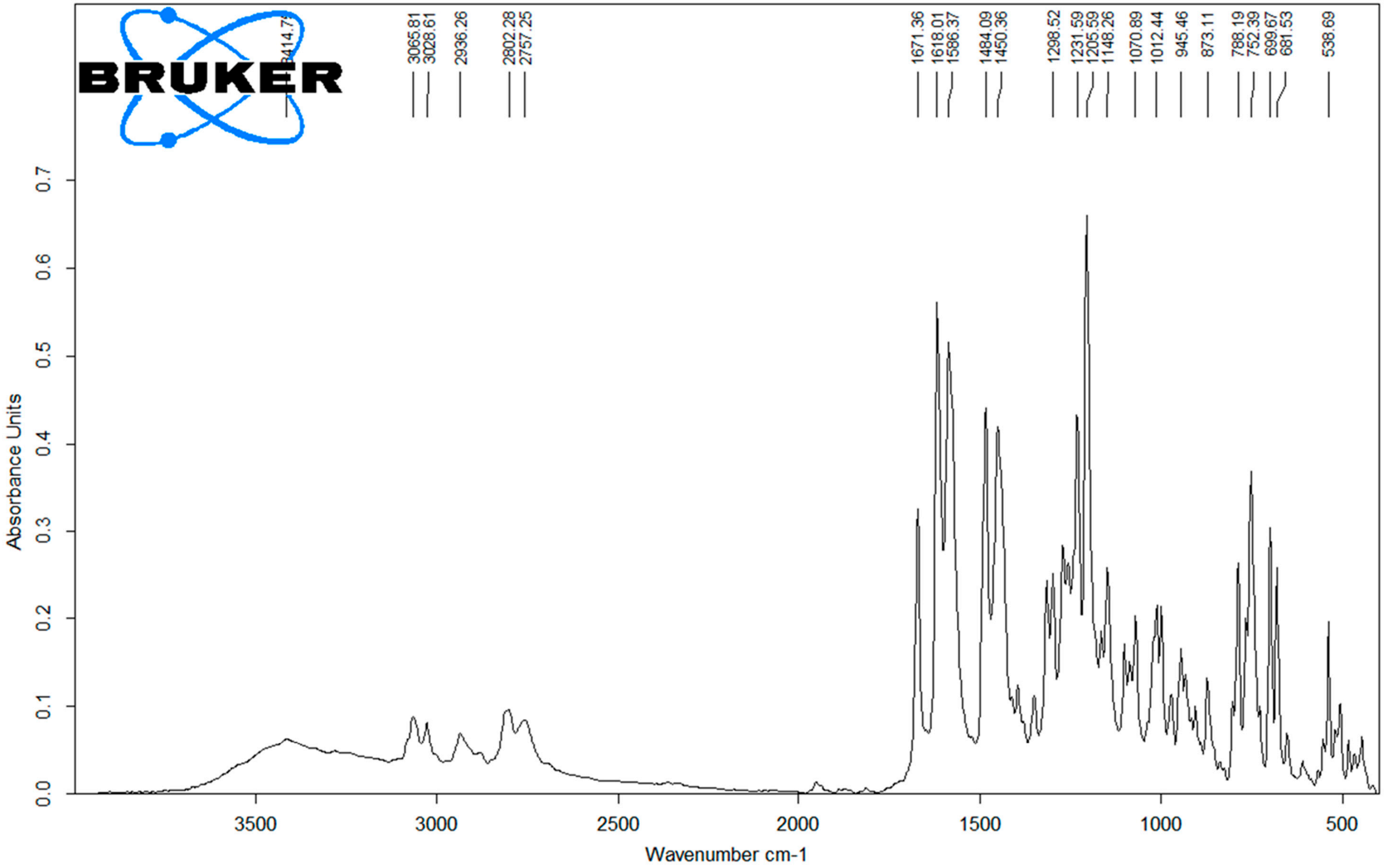

Figure S1. IR (KBr, ν, cm^{-1}) spectrum of 1-benzyl-3,5-bis(2-fluorobenzylidene)piperidone-4 (II).

^{1}H NMR (400 MHz, DMSO-d$_6$, Figure S2):

δ 3.64–3.82 (m, 6H, H_{2ax}, H_{6ax}, H_{2e}q, H_{6e}q, $H_{7,7}$, N-CH_2), 7.00–7.35 (m, 13H, Ar-H, H_9–H_{13}, H_{17}–H_{20}, H_{25}–H_{28}), 7.23 (s, 1H, H_{14}), 7.91 (s, 1H, H_{22}).

Assignment: The multiplet at δ 3.64–3.82 corresponds to the axial and equatorial protons of the piperidine ring (H_{2ax}, H_{6ax}, H_{2e}q, H_{6e}q) and the methylene protons ($H_{7,7}$) of the N-CH_2 group. The aromatic protons from three phenyl rings resonate as a broad multiplet between δ 7.00–7.35, while the two olefinic protons H_{14} and H_{22} appear as singlets at δ 7.23 and 7.91, respectively, consistent with their positions on the conjugated C=C bonds of the dienone system.

^{13}C NMR (100 MHz, DMSO-d$_6$, Figure S3):

δ 187.42 (C_4, C=O), 163.93, 162.23, 161.48, 159.73 (C_{16}, C_{24}, F-substituted Ar-C), 137.36–134.33 (C_3, C_5, C_8, C_{18}, C_{26}, quaternary/aromatic C), 130.19–126.23 (C_9–C_{13}, C_{19}, C_{20}, C_{27}, C_{28}, C_{14}, C_{22}, aromatic and olefinic C), 123.94 (C_{23}), 123.33 (C_{15}), 116.88 (C_{25}), 115.88 (C_{17}), 60.70 (C_7, CH_2), 54.15 (C_2, C_6, piperidine CH_2).

Assignment: The carbonyl carbon (C_4) appears downfield at δ 187.42. Fluorine-substituted aromatic carbons (C_{16}, C_{24}) resonate in the δ 159.73–163.93 region, while quaternary and aromatic carbons (C_3, C_5, C_8, C_{18}, C_{26}) are observed between δ 134.33 and 137.36. Aromatic and olefinic carbons (C_9–C_{13}, C_{19}, C_{20}, C_{27}, C_{28}, C_{14}, C_{22}) occur within δ 126.23–130.19. Additional signals include δ 123.33 (C_{15}), 123.94 (C_{23}), 115.88 (C_{17}), 116.88 (C_{25}), δ 60.70 (C_7, benzylic CH_2), and δ 54.15 (C_2, C_6, piperidine CH_2).

^{2}D NMR Analysis (COSY, HMQC, HMBC):

^{1}H-^{1}H COSY (Figure S4):

Distinct spin–spin couplings were observed, with cross-peaks between the methylene proton H_7 and aromatic protons H_9 and H_{13} at δ 3.81/7.72, and between aromatic protons H_{20} and H_{28} with olefinic protons H_{14} and H_{22} at δ 7.08/7.32. These correlations confirm the expected vicinal proton–proton relationships within the molecular framework.

^{1}H–^{13}C HMQC (Figure S5):

Direct one-bond proton–carbon correlations include H_{2ax}/H_{6ax} and H_{2e}q/H_{6e}q with C_2 and C_6 at δ 3.70–3.75/54.17–54.29, as well as H_{14}/H_{22} with C_{14} and C_{22} at δ 7.19–7.23/135.34–135.35. Additional correlations between aromatic protons and their directly bonded carbons further support the proposed structure.

^{1}H–^{13}C HMBC (Figure S6):

Long-range heteronuclear couplings reveal two- and three-bond correlations. Notably, H_{14} and H_{22} show cross-peaks with C_2 and C_6 at δ 7.73/54.28, confirming the connectivity between the olefinic and piperidine fragments. Extended correlations between aromatic protons and quaternary carbons, including C_4 at δ 187.05–187.42, further substantiate the placement of the carbonyl group within the conjugated system.

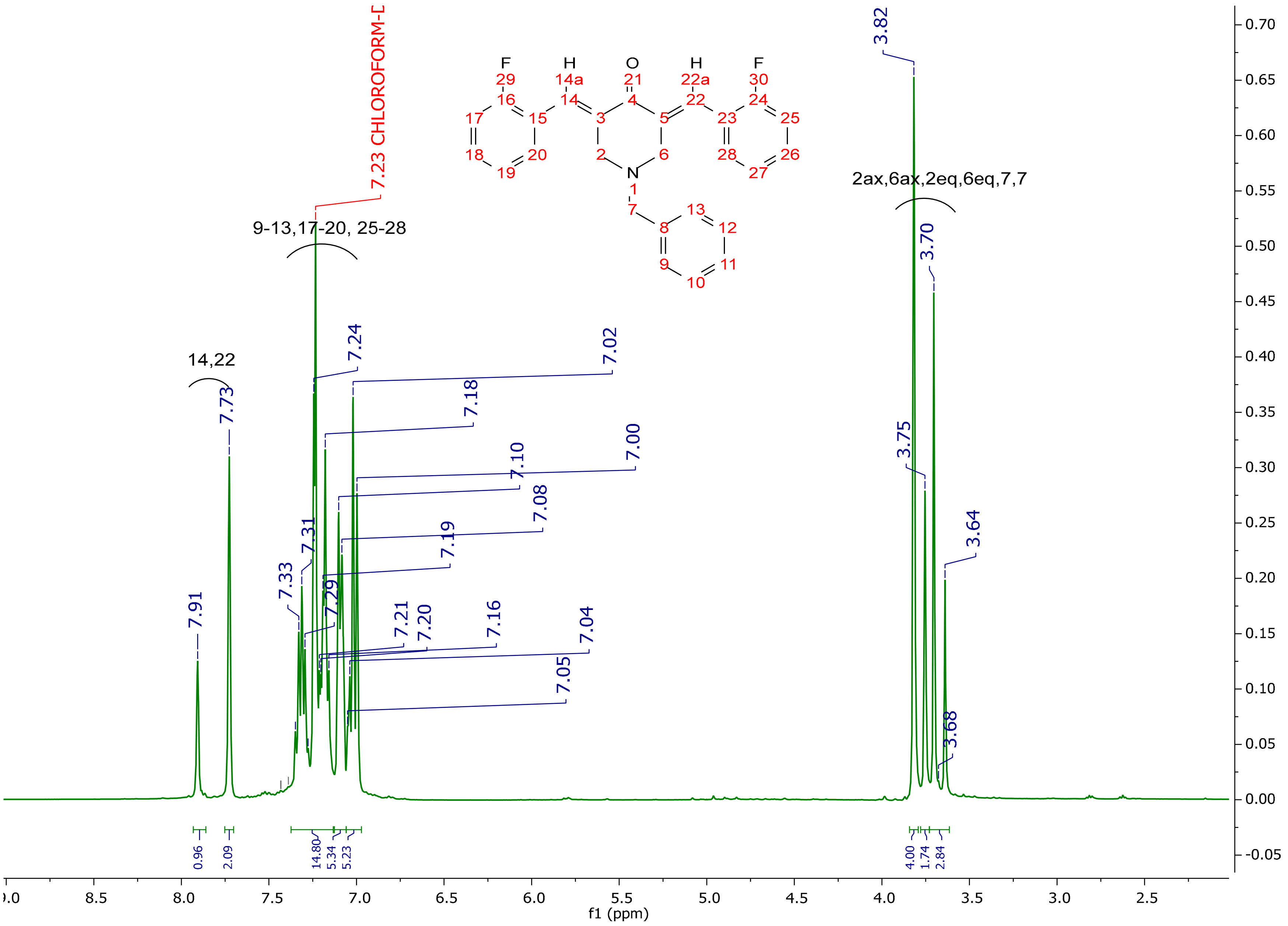

Figure S2. ^{1}H NMR spectrum of 1-benzyl-3,5-bis(2-fluorobenzylidene)piperidone-4 (II) in $CDCl_3$.

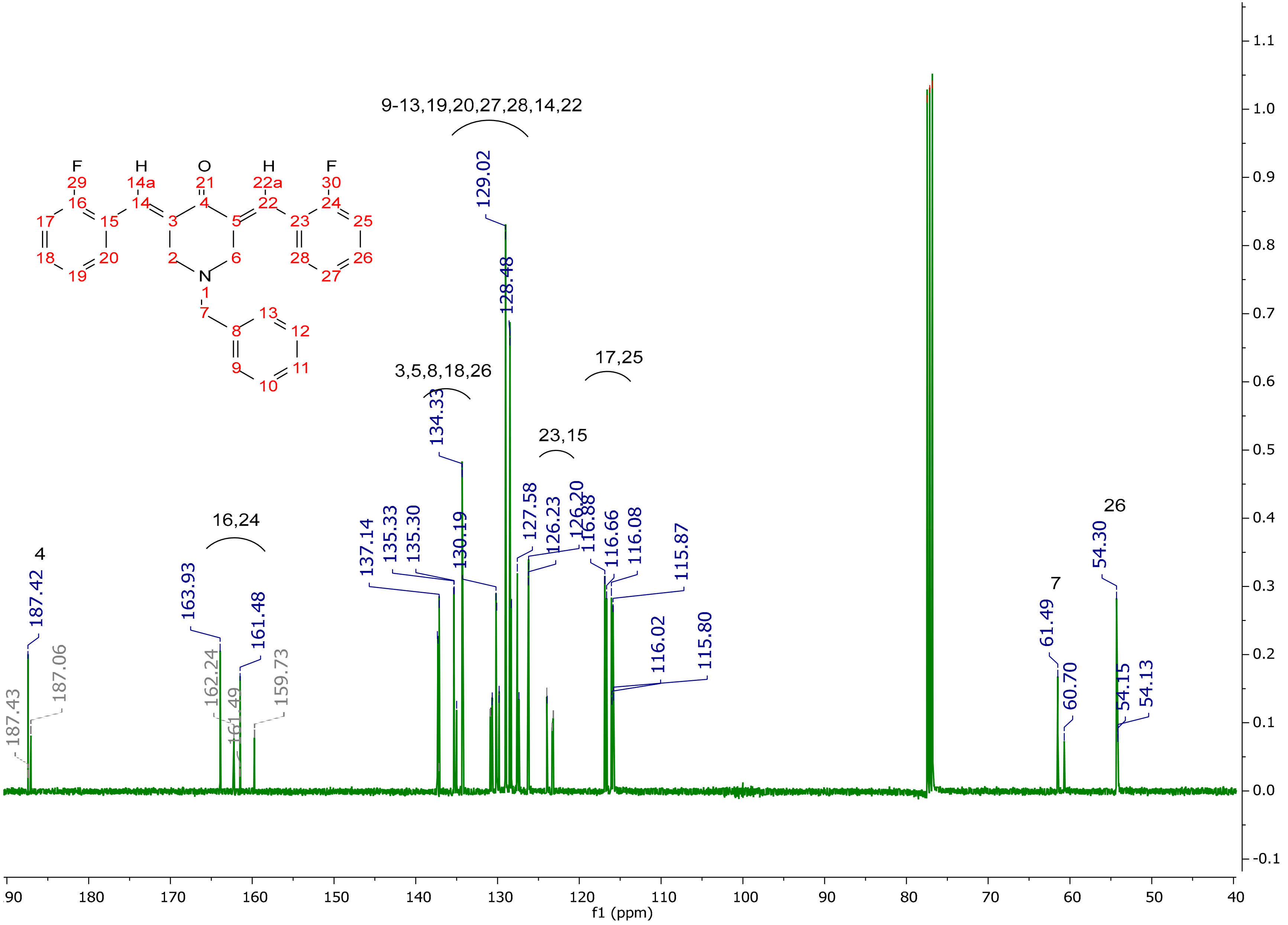

Figure S3. ^{13}C NMR spectrum of 1-benzyl-3,5-bis(2-fluorobenzylidene)piperidone-4 (II) in $CDCl_3$.

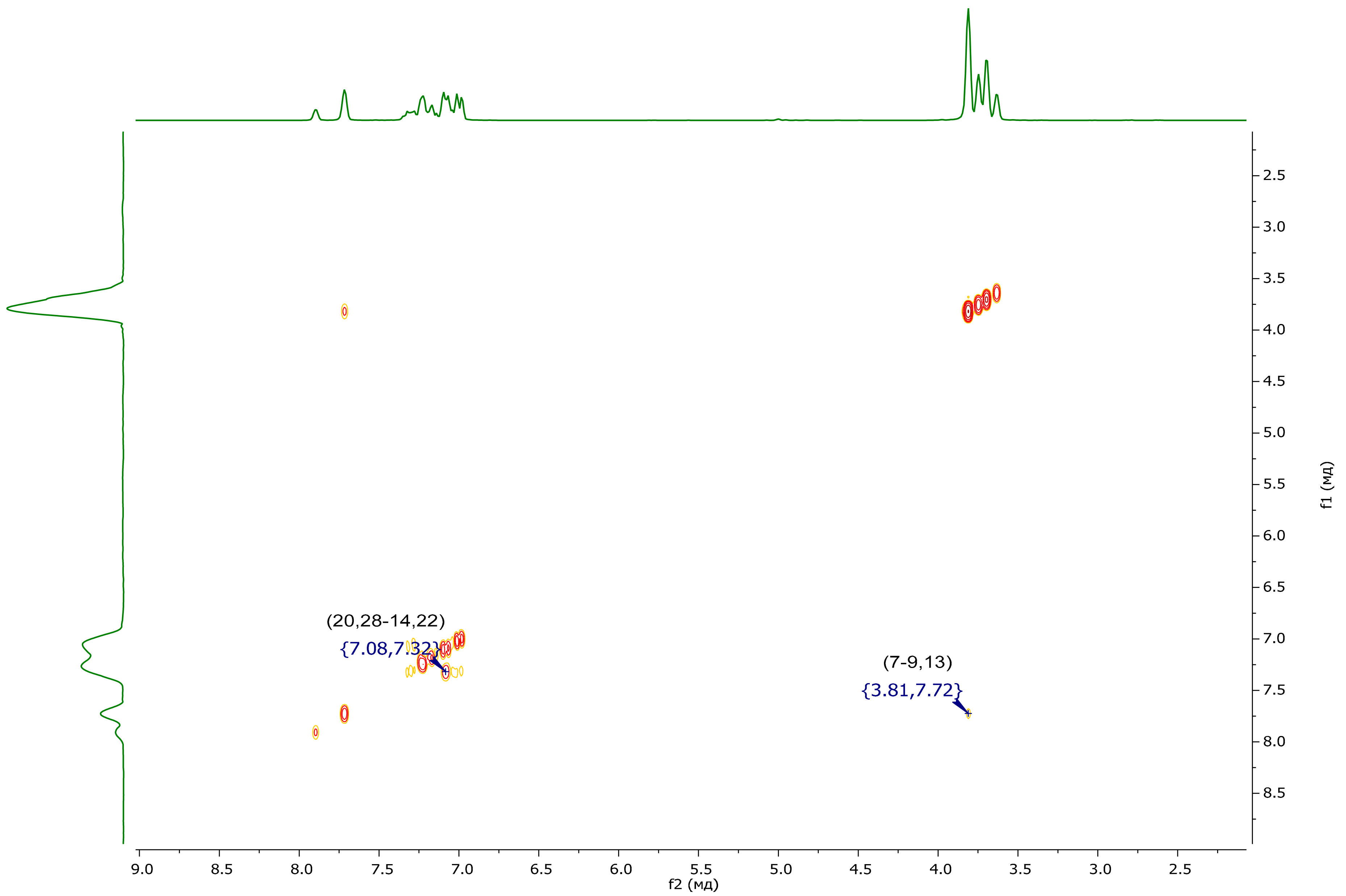

Figure S4. NMR ^{1}H-^{1}H COSY NMR spectrum of 1-benzyl-3,5-bis(2-fluorobenzylidene)piperidone-4 (II) in $CDCl_3$.

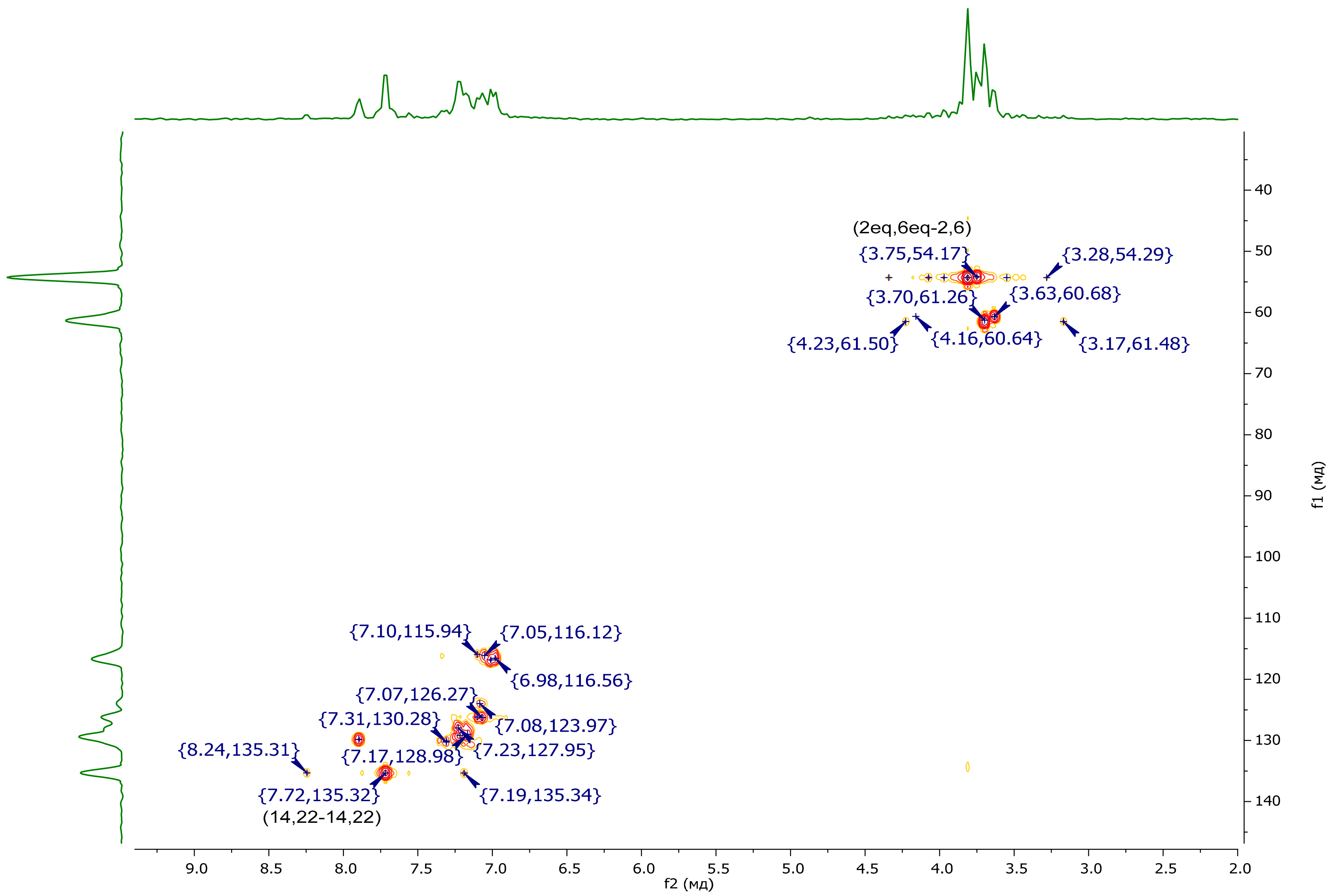

Figure S5. NMR ^{1}H-^{13}C HMQC NMR spectrum of 1-benzyl-3,5-bis(2-fluorobenzylidene)piperidone-4 (II) in $CDCl_3$.

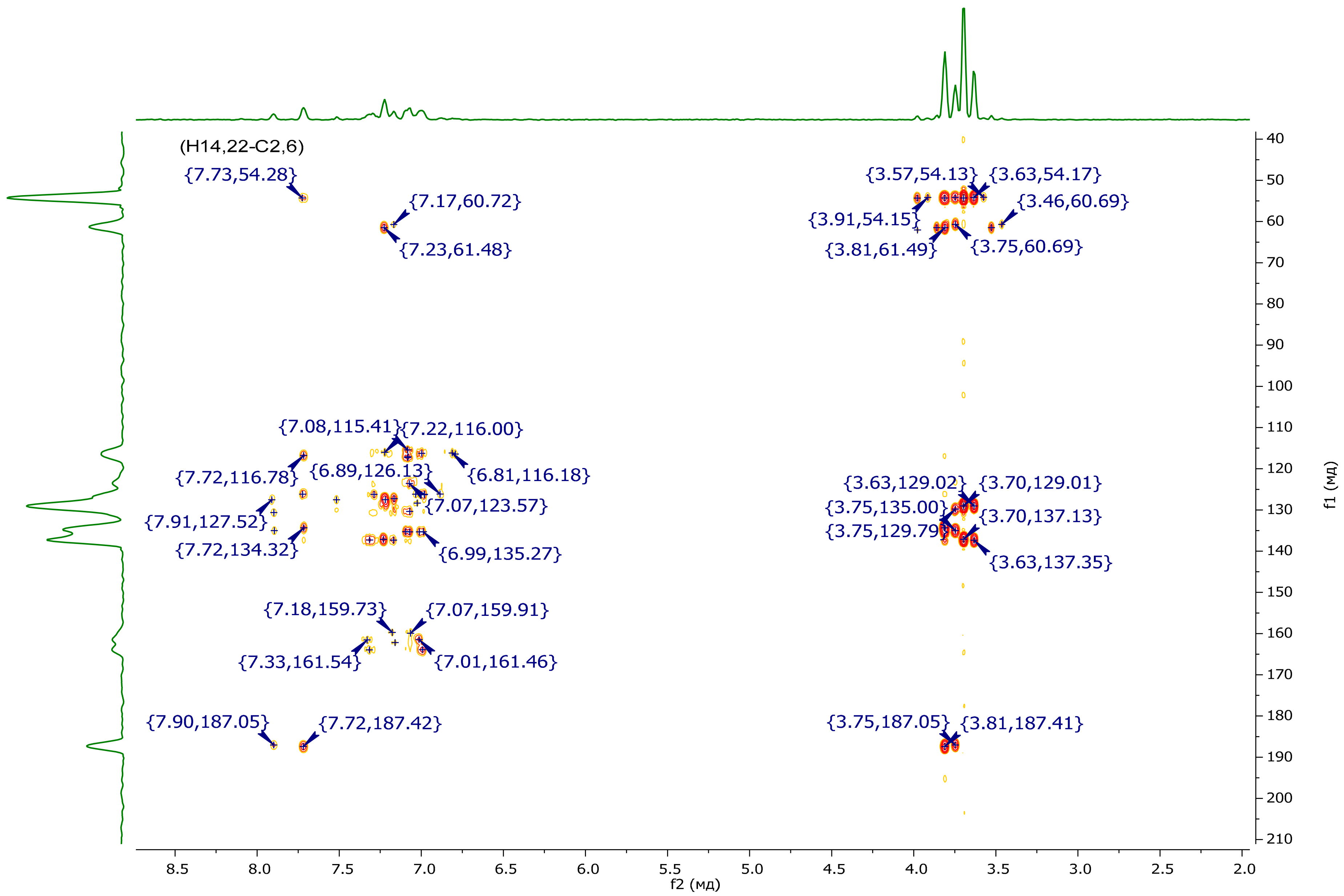

Figure S6. NMR ^{1}H-^{13}C HMBC NMR spectrum of 1-benzyl-3,5-bis(2-fluorobenzylidene)piperidone-4 (II) in $CDCl_3$.

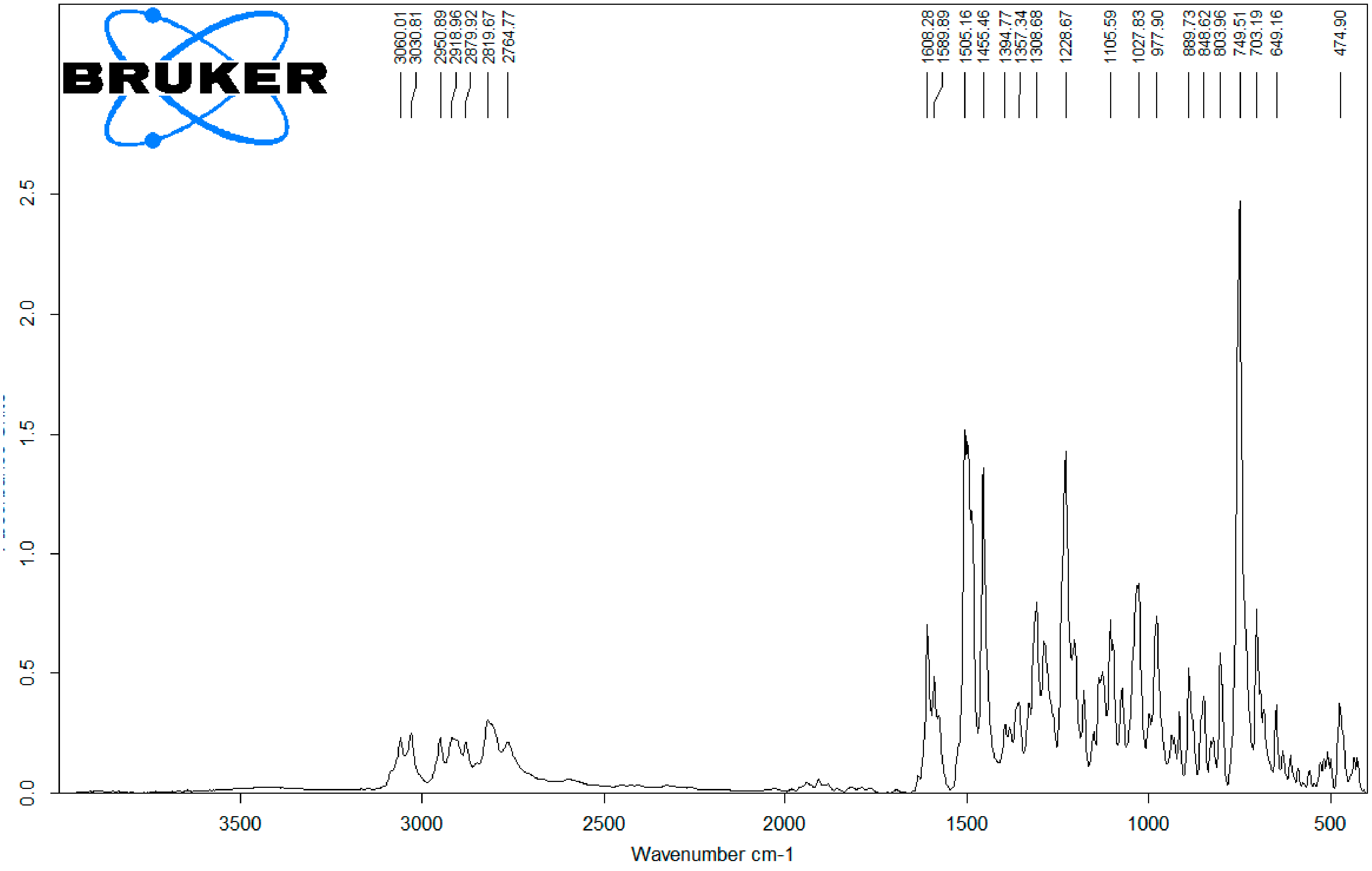

Figure S7. IR (KBr, ν, cm^{-1}) spectrum of 5-benzyl-7-(2-fluorobenzylidene)-2,3-bis(*o*-fluorophenyl)-3,3a,4,5,6,7-hexahydro-2H-pyrazolo[4,3-*c*]pyridine (PP).

^{1}H NMR (400 MHz, DMSO-d$_6$, Figure S8):

δ 2.63–2.66 (m, 1H, H_{2ax}), 3.36–3.43 (m, 1H, H_{2e}q), 3.23 (d, 1H, H_{9ax}, ^2J = 14.2 Hz), 3.92 (d, 1H, H_{9e}q, ^2J = 14.2 Hz), 3.43–3.50 (m, 1H, H_3), 3.64–3.73 (m, 2H, $H_{10/10}$), 5.28 (d, 1H, H_4, ^3J = 12.8 Hz), 6.98–7.16 (m, 9H, H_{29}, H_{32}, H_{25}, H_{13}, H_{14}, H_{15}, H_{34}, H_{28}, H_{26}), 7.17–7.27 (m, 7H, H_{12}, H_{16}, H_{35}, H_{27}, H_{19}, H_{20}, H_{21}), 7.38 (s, 1H, H_{22}), 7.62–7.65 (m, 1H, H_{33}).

Assignment: The piperidine protons are clearly resolved, with H_{2ax} and H_{2e}q appearing as multiplets at δ 2.63–2.66 and 3.36–3.43, respectively. The geminal protons at C_9 (H_{9ax} and H_{9e}q) are observed as doublets at δ 3.23 and 3.92 (^2J = 14.2 Hz). Proton H_3 resonates as a multiplet at δ 3.43–3.50, while the methylene protons at C_{10} appear as a two-proton multiplet at δ 3.64–3.73. The pyrazole H_4 signal is a doublet at δ 5.28 (^3J = 12.8 Hz). The aromatic region exhibits characteristic signals, including multiplets at δ 6.98–7.16 (9H) and 7.17–7.27 (7H), a singlet at δ 7.38 (H_{22}), and a multiplet at δ 7.62–7.65 (H_{33}), consistent with the four aromatic rings and the olefinic environment.

^{13}C NMR (100 MHz, DMSO-d$_6$, Figure S9):

δ 161.97 (C_7, quaternary C), 159.49 (C_{31}), 156.49 (C_{24}), 153.49 (C_{18}), 152.79 (C_8), 137.62 (C_{11}), 133.93 (C_{33}), 130.83 (C_{30}), 130.41 (C_{20}), 129.41 (C_{14}), 129.20 (C_{12}, C_{16}), 129.04 (C_{13}, C_{15}), 127.37 (C_{22}, C_{26}, C_{27}), 126.76 (C_{23}), 124.82 (C_{21}), 123.94 (C_{34}), 123.11 (C_{35}), 120.79 (C_{29}, C_{28}), 119.59 (C_{17}), 116.53 (C_{19}), 115.42 (C_{32}), 114.57 (C_{25}), 65.78 (C_4, pyrazole C), 61.76 (C_{10}, CH_2), 55.48 (C_2), 54.96 (C_9), 53.66 (C_3).

Assignment: The piperidine ring carbons are observed at δ 53.66 (C_3), 54.96 (C_9), and 55.48 (C_2), while quaternary carbons within the nitrogen-containing ring resonate at δ 152.79 (C_8) and 161.97 (C_7). The fused pyrazole carbon C_4 appears at δ 65.78, and the methylene carbon C_{10} at δ 61.76. The olefinic carbon C_{29}, overlapping with aromatic carbon C_{28}, is detected at δ 120.79. Aromatic carbons resonate between δ 114.57 and 137.62, and additional quaternary carbons are present at δ 153.49 (C_{18}), 156.49 (C_{24}), and 159.49 (C_{31}).

^{1}H–^{1}H COSY (Figure S10):

δ 2.65/3.46 (H_{2ax}–H_3), 3.38/3.89 (H_{2e}q–H_{9e}q), 3.42/5.25 (H_3–H_4), 7.03/7.27 (H_{32}–H_{33}).

Assignment: The observed three-bond cross-peaks confirm scalar couplings between vicinal methylene and methine protons within the piperidine ring, as well as between aromatic protons. These correlations establish the expected connectivity across both the aliphatic and aromatic regions of the molecule.

^{1}H–^{13}C HMQC (Figure S11):

δ 2.63/55.43 (H_{2ax}–C_2), 3.20/54.38 (H_{2e}q–C_2), 3.41/53.67 (H_{9ax}–C_9), 3.47/53.37 (H_{9e}q–C_9), 3.68/61.65 (H_{10}–C_{10}), 3.64/61.72 (H_3–C_3), 5.25/65.69 (H_4–C_4), 7.24/129.15 ($H_{12/16}$–$C_{12/16}$), 7.05/123.71 (H_{29}–C_{29}), 6.98/115.33 (H_{32}–C_{32}), 7.01/115.43 (H_{25}–C_{25}).

Assignment: The observed one-bond proton–carbon cross-peaks confirm direct connectivities throughout the molecule, including characteristic correlations within the piperidine ring, fused pyrazole unit, and aromatic moieties. These results support the structural integrity and assignment of all key proton–carbon pairs in PP.

^{1}H–^{13}C HMBC (Figure S12):

δ 2.46/65.58 (H_{2ax}–C_4), 3.88/55.43 (H_{9e}q–C_2), 3.81/61.76 (H_{9e}q–C_{10}), 3.61/129.07 (H_{10}–C_{12}/C_{16}), 5.24/53.63 (H_4–C_2), 5.24/128.10 (H_4–C_{22}), 5.26/159.36 (H_4–C_7), 7.60/159.54 (H_{33}–C_{31}), 7.34/152.72 (H_{33}–C_7).

Assignment: The long-range proton–carbon correlations establish key structural linkages across the pyrazolopiperidine framework. Interactions involving H_{2ax}, H_{9e}q, and H_4 confirm connectivity between the aliphatic piperidine core and the fused heterocyclic and aromatic regions, while cross-peaks from H_{33} to quaternary carbons provide additional evidence for the spatial arrangement of aromatic substituents.

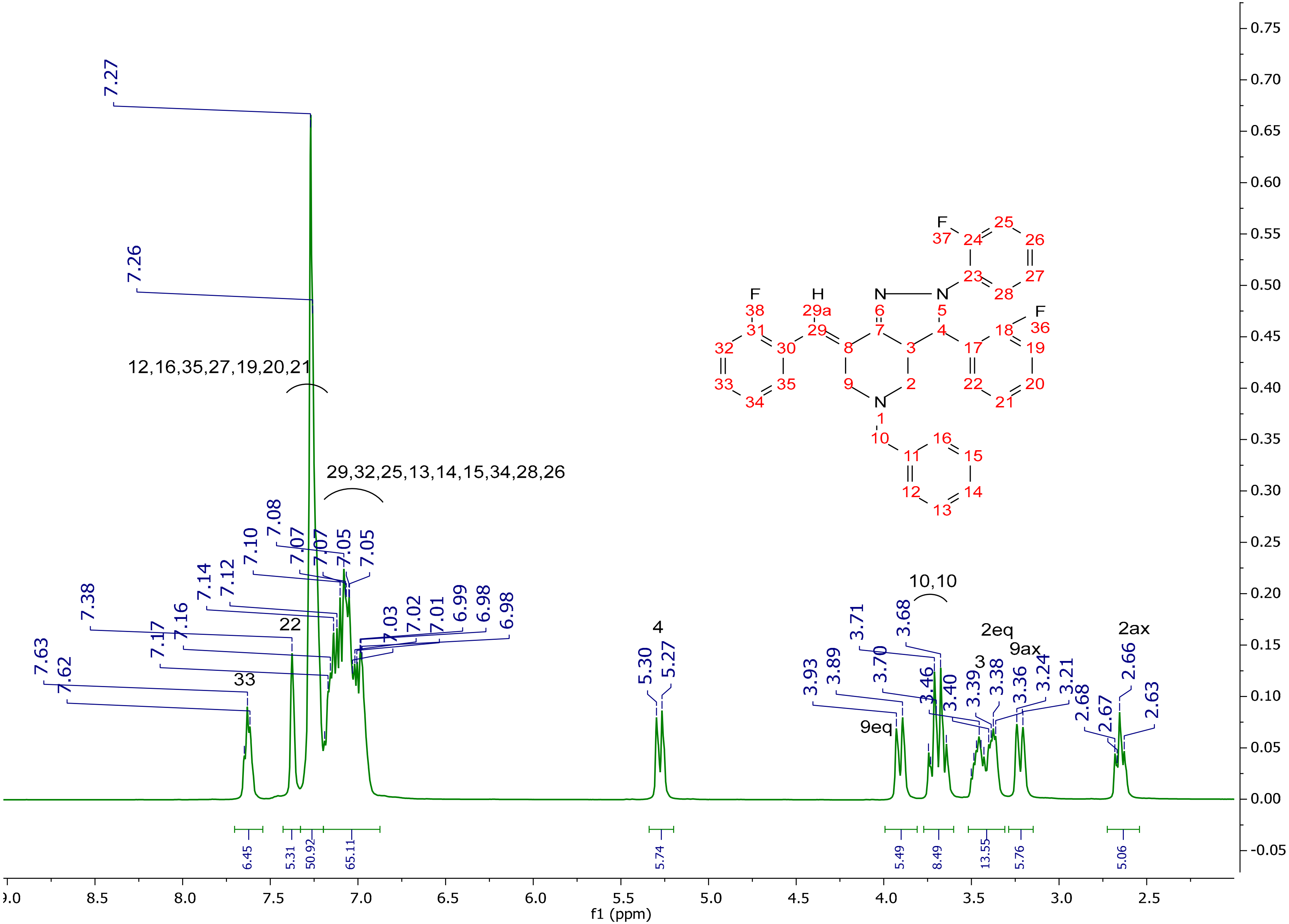

Figure S8. ^{1}H NMR spectrum of 5-benzyl-7-(2-fluorobenzylidene)-2,3-bis(2-fluorophenyl)-3,3a,4,5,6,7-hexahydro-2H-pyrazolo[4,3-*c*]pyridine (PP) in $CDCl_3$.

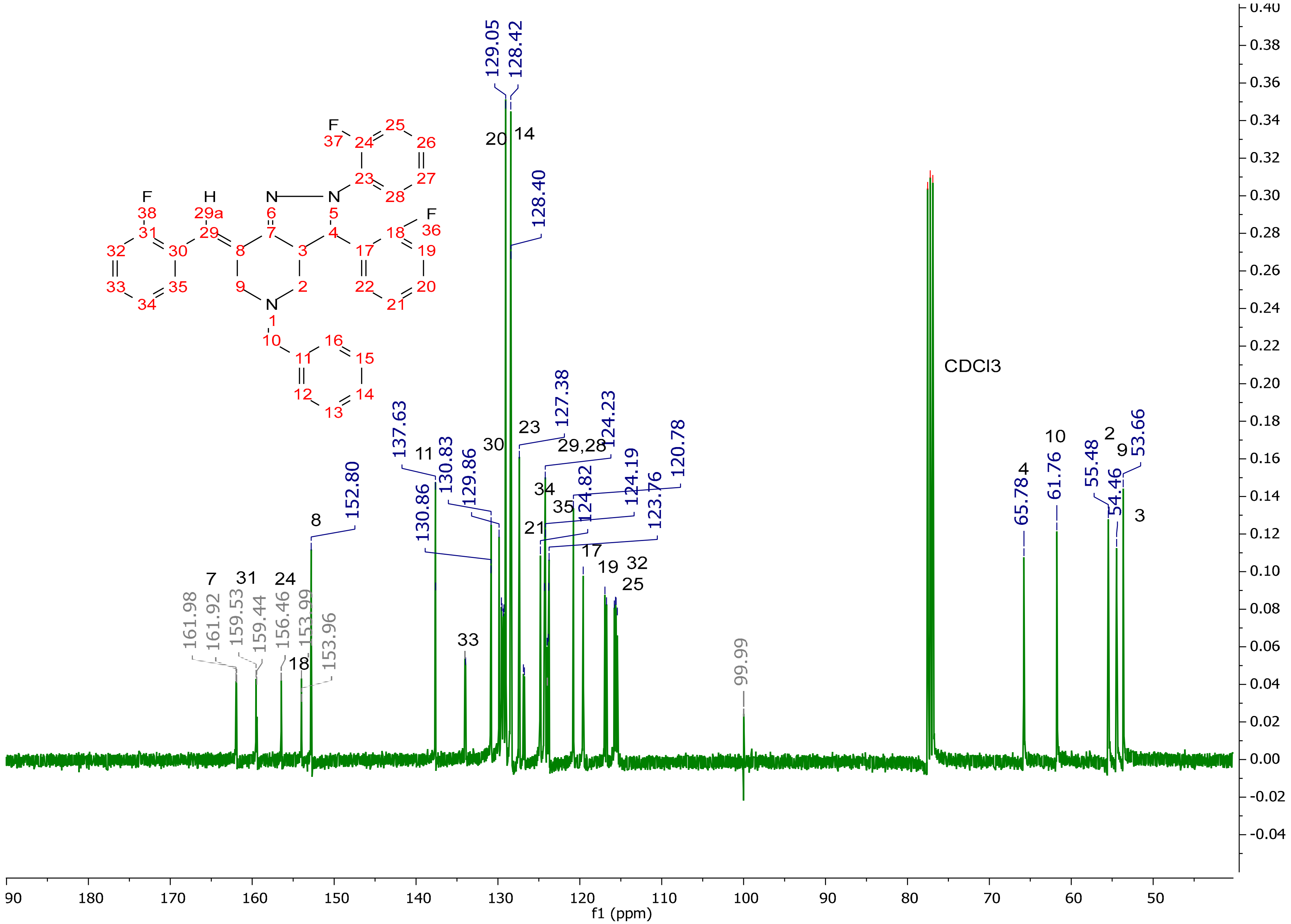

Figure S9. ^{13}C NMR spectrum of 5-benzyl-7-(2-fluorobenzylidene)-2,3-bis(2-fluorophenyl)-3,3a,4,5,6,7-hexahydro-2H-pyrazolo[4,3-*c*]pyridine (PP) in $CDCl_3$.

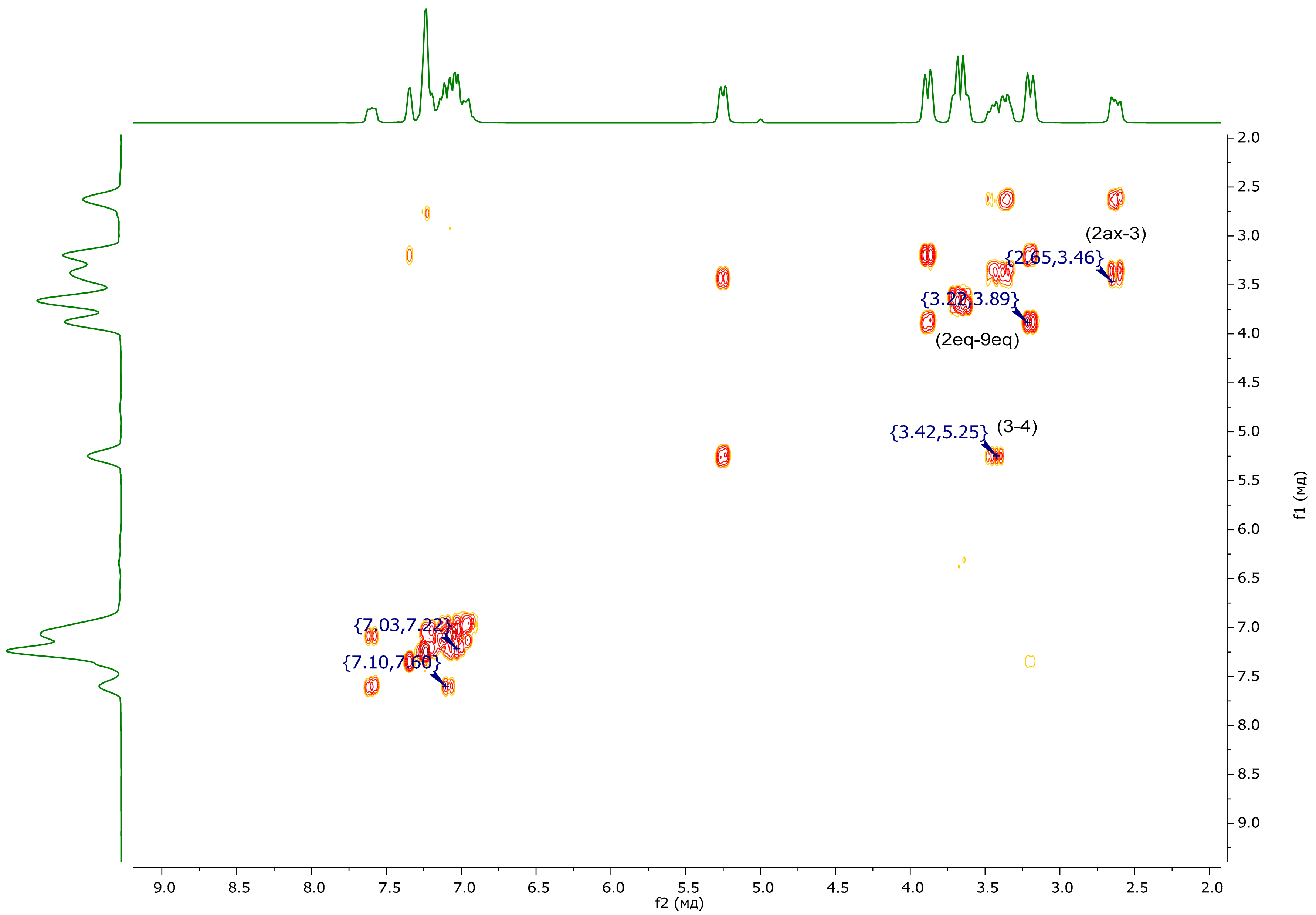

Figure S10. NMR ^{1}H-^{1}H COSY NMR spectrum of 5-benzyl-7-(2-fluorobenzylidene)-2,3-bis(2-fluorophenyl)-3,3a,4,5,6,7-hexahydro-2H-pyrazolo[4,3-*c*]pyridine (PP) in $CDCl_3$.

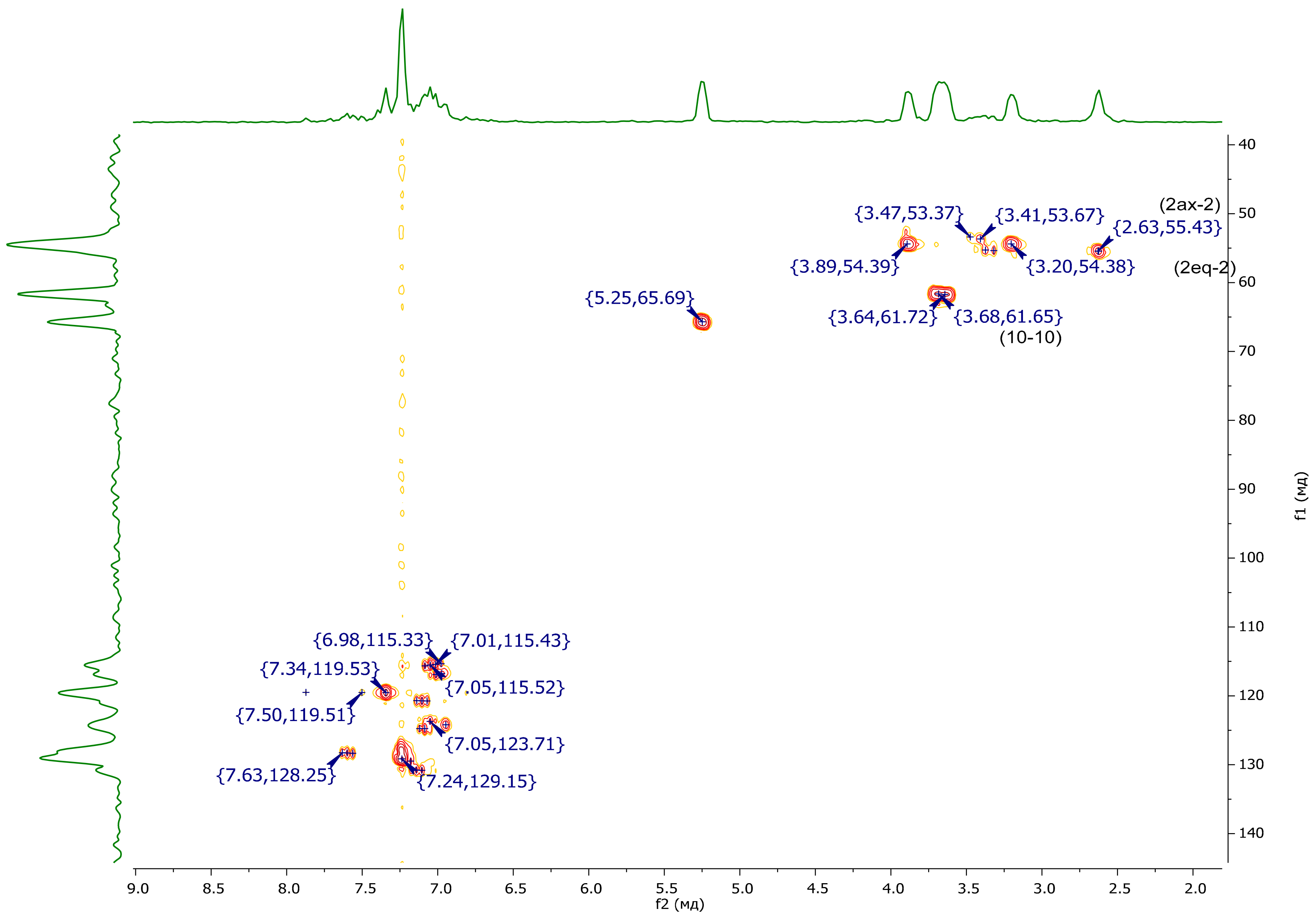

Figure S11. NMR ^{1}H-^{13}C HMQC NMR spectrum of 5-benzyl-7-(2-fluorobenzylidene)-2,3-bis(2-fluorophenyl)-3,3a,4,5,6,7-hexahydro-2H-pyrazolo[4,3-*c*]pyridine (PP) in $CDCl_3$.

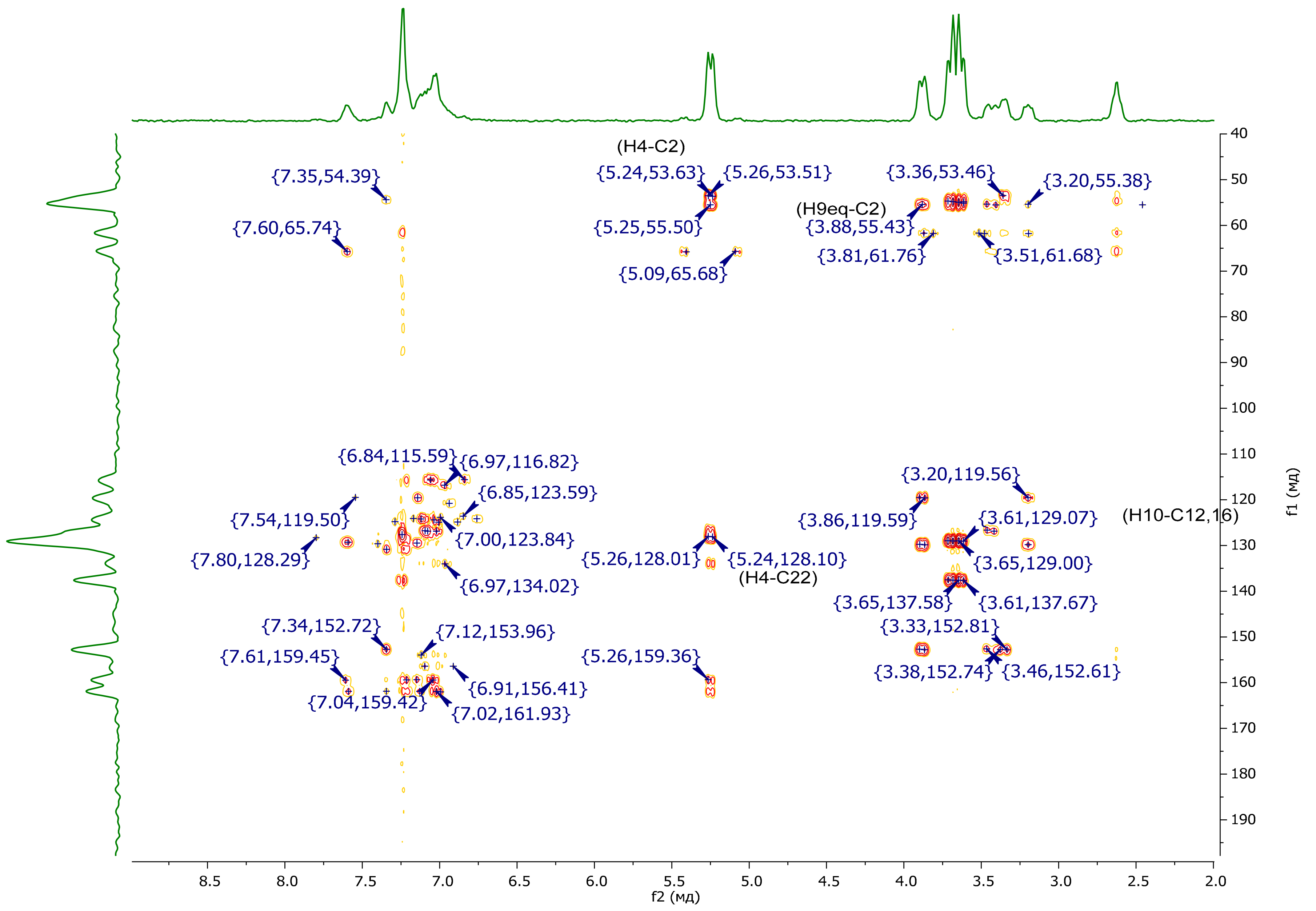

Figure S12. NMR ^{1}H-^{13}C HMBC NMR spectrum of 5-benzyl-7-(2-fluorobenzylidene)-2,3-bis(2-fluorophenyl)-3,3a,4,5,6,7-hexahydro-2H-pyrazolo[4,3-*c*]pyridine (PP) in $CDCl_3$.

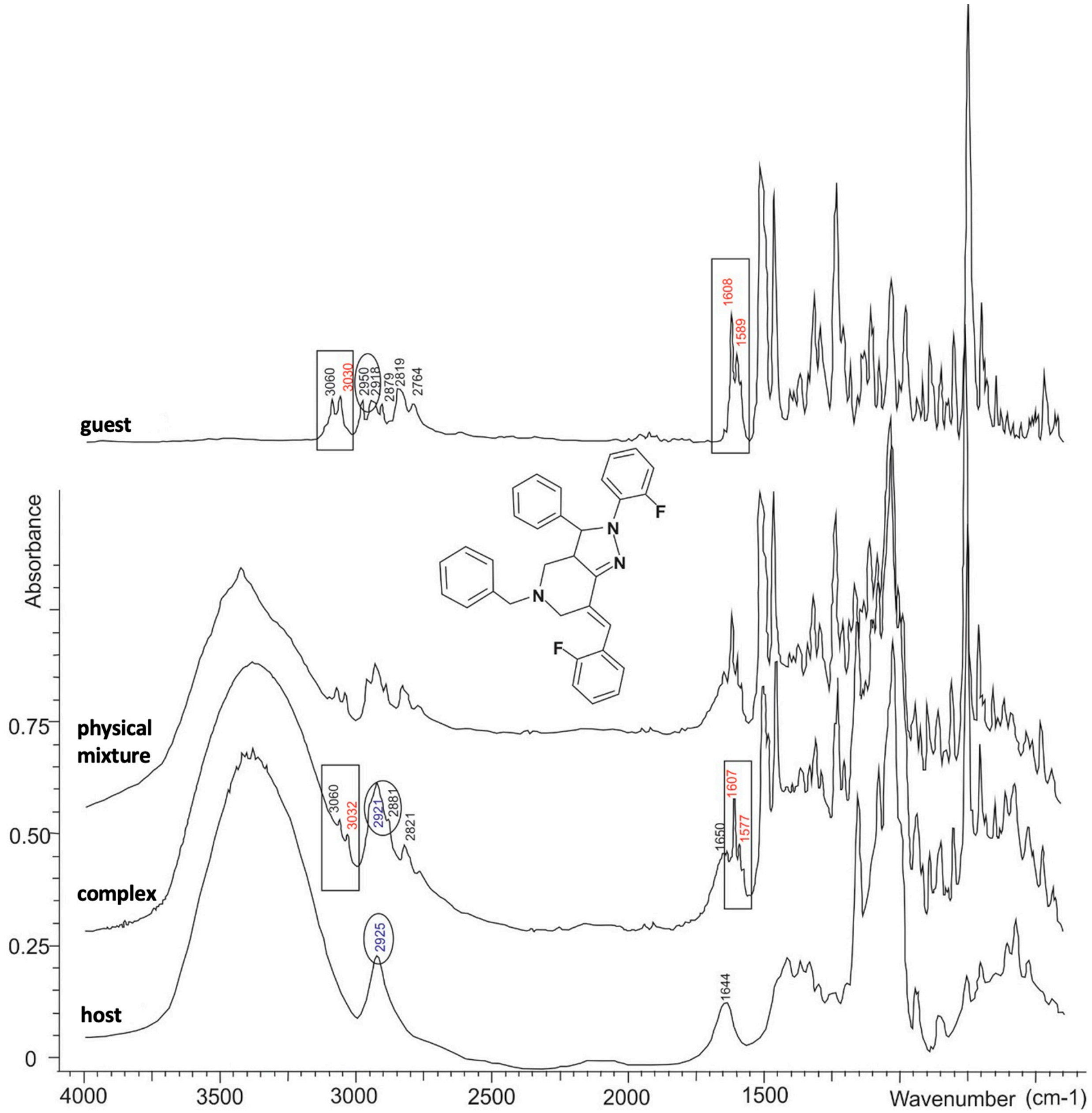

Figure S13. IR (KBr, ν, cm^{-1}) spectrum of the physical mixture (1:1), complex (**PPβCD**) and the starting components (PP and *β*-cyclodextrin).

^{1}H NMR (Figure S14):

δ 2.46 (m, H_{2ax}), 3.25–3.27 (m, H_{2e}q), 3.20 (m, H_{9ax}), 3.47 (m, H_3), 3.41–3.59 (m, H_{9e}q + $H_{10/10}$, overlapping with β-CD signals), 3.65 (m, $H_{10/10}$), 4.55–4.78 (d, J ≈ 13.4 Hz, H_4), 7.00–7.40 (m, aromatic protons $H_{12–16}$, H_{32}, H_{34}, H_{35}, $H_{25–28}$, $H_{19–22}$, H_{29}), 7.52 (s, H_{33}).

Assignment: The proton spectrum of the PPβCD complex shows well-resolved signals for the aliphatic piperidine protons, with characteristic shifts indicative of inclusion within the β-cyclodextrin cavity. Overlapping resonances in the 3.4–3.6 ppm region reflect host–guest interactions. The aromatic and olefinic regions remain consistent with the free compound, confirming structural integrity upon encapsulation.

^{1}H NMR (β-CD signals in PPβCD, Figure S14):

δ 3.25–3.28 (m, H_2), 3.28–3.32 (m, H_4), 3.50–3.54 (m, H_5, H_3, H_6), 4.78 (s, H_1).

Assignment: The oligosaccharide region of β-cyclodextrin displays characteristic multiplets for the internal ring protons, with H_2 and H_4 resolved in the low-field region and a combined multiplet for H_5, H_3, and H_6. The anomeric proton H_1 appears as a sharp singlet, confirming the structural integrity of the β-CD host in the inclusion complex.

^{13}C NMR (PPβCD, Figure S15):

δ 52.83 (C_3), 53.86 (C_9), 54.54 (C_2), 61.00 (C_{10}), 66.19 (C_4), 116.07 (C_{25}, C_{32}), 117.05 (C_{19}), 121.04 (C_{28}, C_{29}), 124.29 (C_{35}), 127.68 (C_{22}, C_{27}, C_{28}), 128.71 (C_{13}, C_{15}), 129.36 (C_{12}, C_{16}), 129.93 (C_{20}), 131.13 (C_{30}), 134.02 (C_{33}), 138.22 (C_{11}), 152.62 (C_8), 154.62 (C_{18}), 159.89 (C_{24}), 161.52 (C_7).

Assignment: The carbon signals of the piperidine moiety are clearly resolved in the low-field region, with the heterocyclic C_4 and methylene C_{10} appearing at δ 66.19 and 61.00 ppm, respectively. Aromatic carbons and the olefinic C_{29} resonate across δ 116.07–138.22 ppm, while quaternary and fluorine-substituted carbons are observed downfield, reflecting the electronic influence of substitution and inclusion complex formation.

^{13}C NMR (β-CD moiety in PPβCD):

δ 60.22 (C_6), 72.53 (C_5), 73.55 (C_3), 73.86 (C_2), 82.01 (C_4), 102.44 (C_1).

Assignment: The characteristic carbon resonances of the β-cyclodextrin oligosaccharide framework are clearly detected, with primary and secondary hydroxyl-bearing carbons appearing in the δ 60–82 ppm range and the anomeric carbon (C_1) resonating at δ 102.44 ppm, confirming the presence and structural integrity of the β-CD host within the inclusion complex.

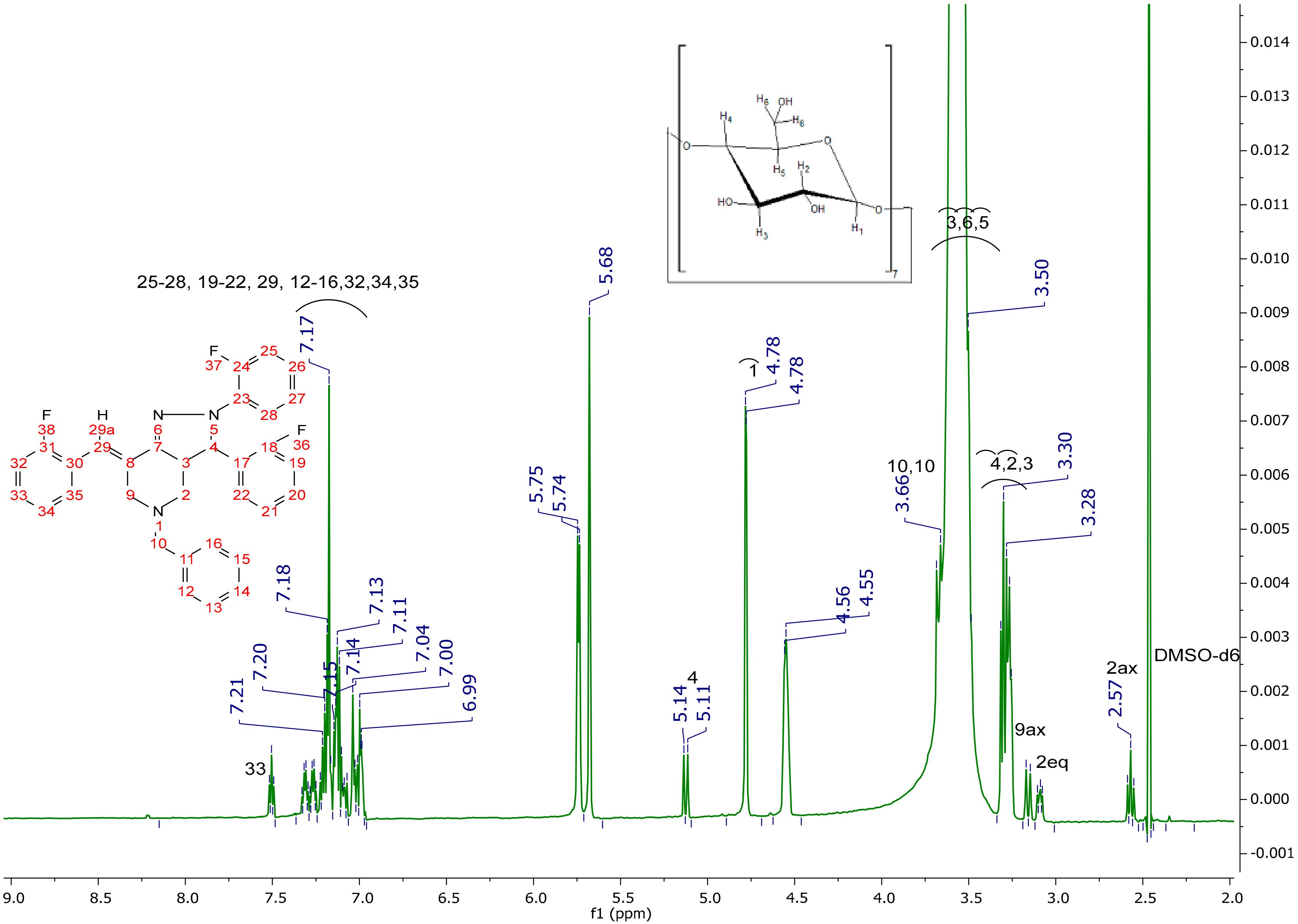

Figure S14. ^{1}H NMR spectrum of Complex of 5-benzyl-7-(2-fluorobenzylidene)-2,3-bis(2-fluorophenyl)-3,3a,4,5,6,7-hexahydro-2H-pyrazolo[4,3-*c*]pyridine with β-CD (**PPβCD)** in DMSO-d_6.

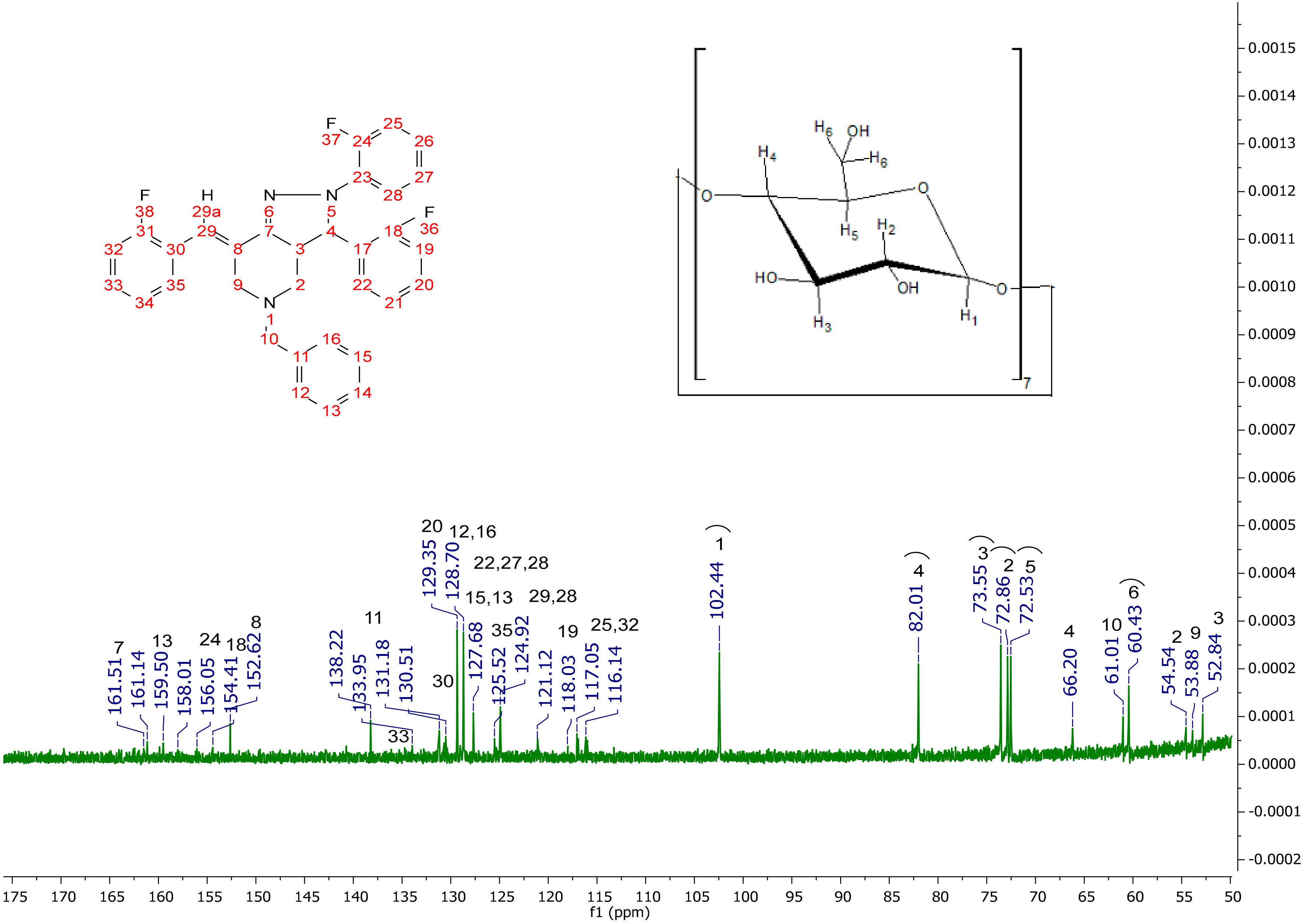

Figure S15. ^{13}C NMR spectrum of Complex of 5-benzyl-7-(2-fluorobenzylidene)-2,3-bis(2-fluorophenyl)-3,3a,4,5,6,7-hexahydro-2H-pyrazolo[4,3-*c*]pyridine with β-CD (**PPβCD)** in DMSO-d$_6$.

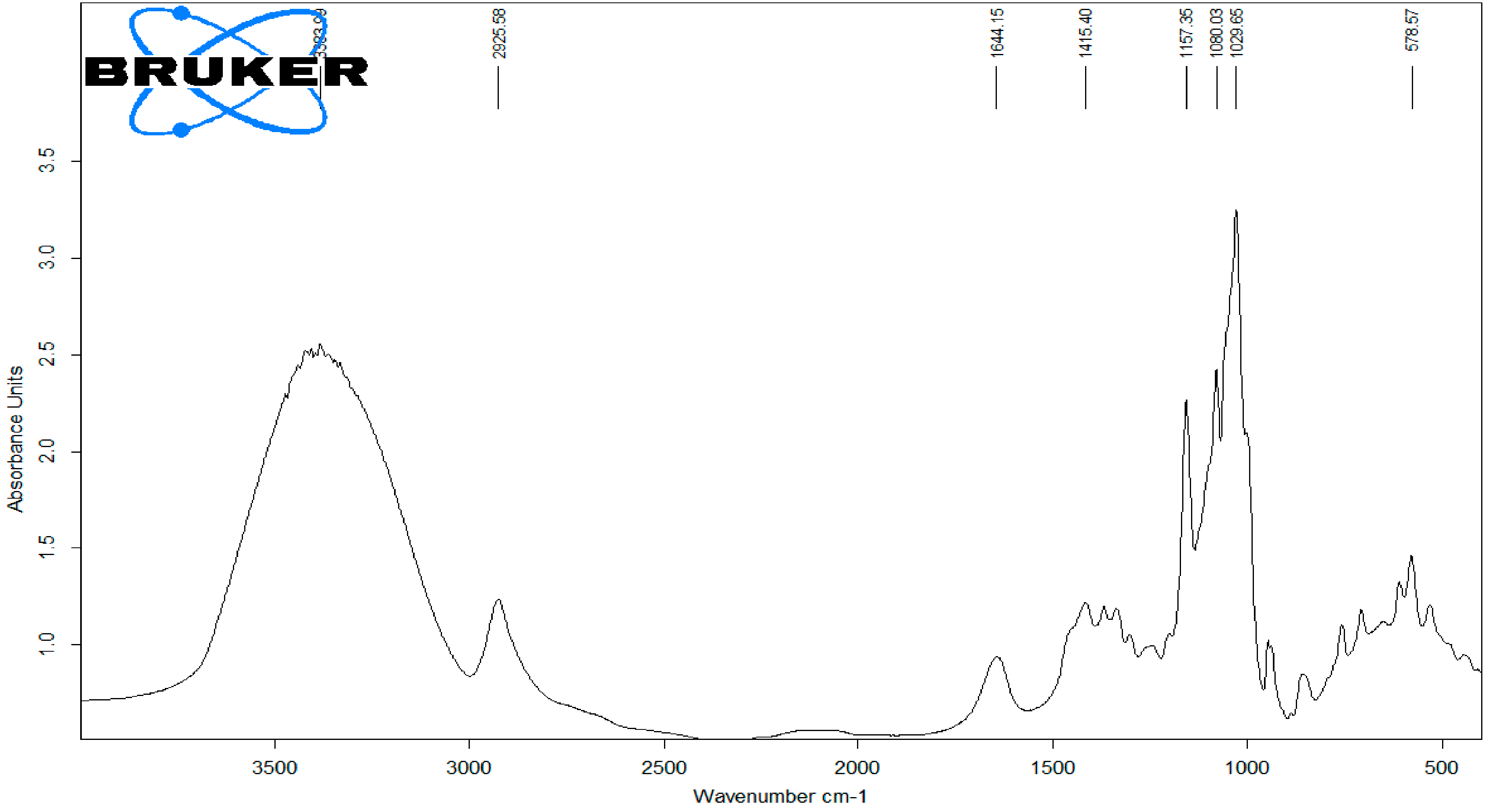

Figure S16. IR (KBr, ν, cm^{-1}) spectrum of *β*-cyclodextrin.

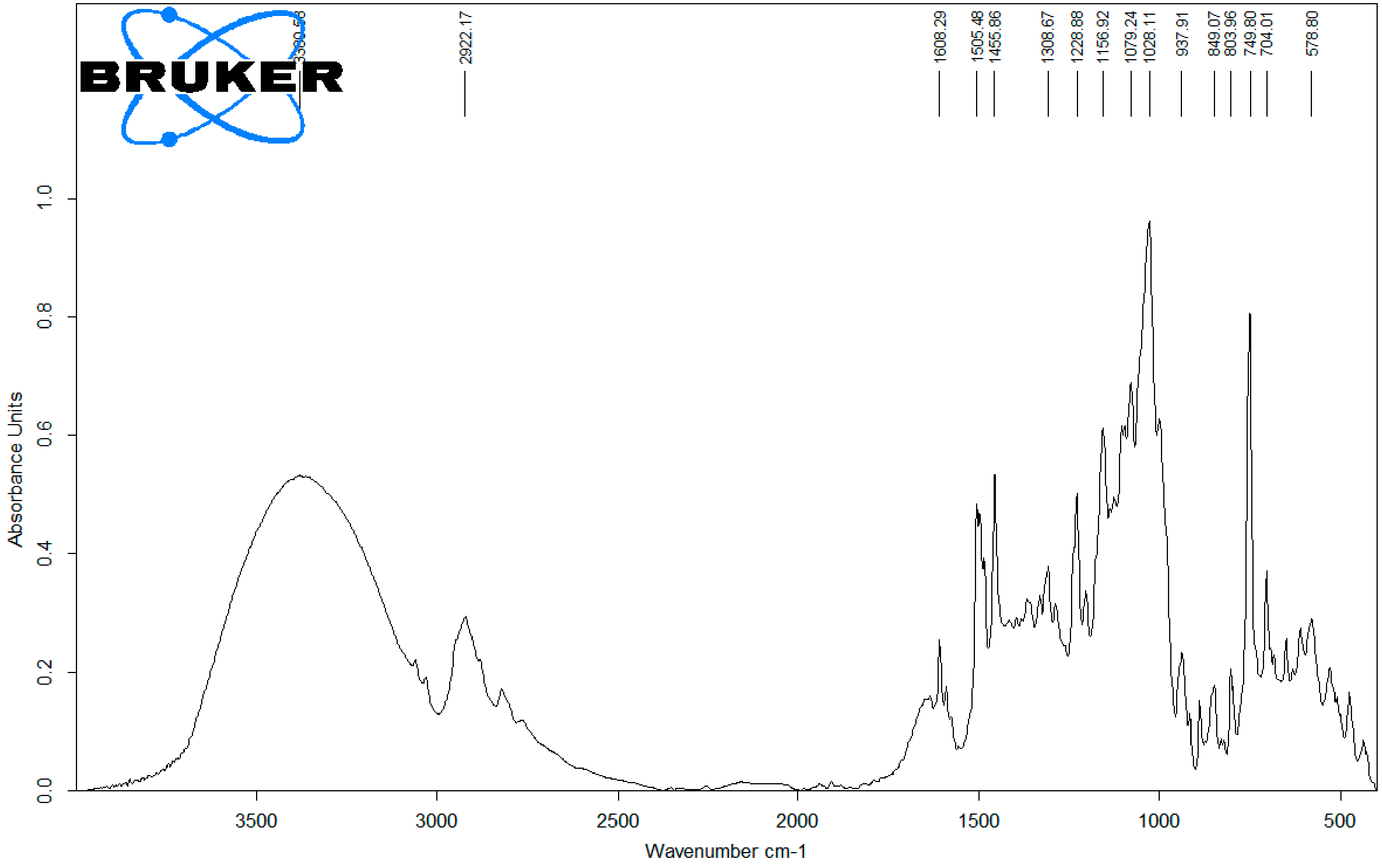

Figure S17. IR (KBr, ν, cm^{-1}) spectrum of *β*-cyclodextrin with 5-benzyl-7-(2-fluorobenzylidene)-2,3-bis(2-fluorophenyl)-3,3a,4,5,6,7-hexahydro-2H-pyrazolo[4,3-*c*]pyridine (**PPβCD**).
